# Selection of oxygen reduction catalysts for secondary tri-electrode zinc–air batteries

**DOI:** 10.1038/s41598-022-10671-5

**Published:** 2022-04-23

**Authors:** Adeline Loh, David P. Trudgeon, Xiaohong Li, Mao-Cheng Liu, Ling-Bin Kong, Frank C. Walsh

**Affiliations:** 1 Renewable Energy Group, College of Engineering, Mathematics and Physical Sciences, University of Exeter, Penryn Campus, Cornwall, TR10 9FE UK; 2 State Key Laboratory of Advanced Processing and Recycling of Non-Ferrous Metals, School of Materials Science and Engineering, Lanzhou University of Technology, Lanzhou, 730050 China; 3 Electrochemical Engineering Laboratory, Energy Technology Group, Department of Mechanical Engineering, University of Southampton, Southampton, SO17 1BJ UK

**Keywords:** Chemistry, Energy science and technology, Engineering, Materials science

## Abstract

Oxygen reduction reaction (ORR) electrocatalysts, which are highly efficient, low-cost, yet durable, are important for secondary Zn–air cell applications. ORR activities of single and mixed metal oxide and carbon electrocatalysts were studied using rotating disc electrode (RDE) measurements, Tafel slope and Koutecky–Levich plots. It was found that MnO_x_ combined with XC-72R demonstrated high ORR activity and good stability—up to 100 mA cm^−2^. The performance of the selected ORR electrode and a previously optimised oxygen evolution reaction (OER) electrode was thereafter tested in a custom-built secondary Zn–air cell in a tri-electrode configuration, and the effects of current density, electrolyte molarity, temperature, and oxygen purity on the performance of the ORR and OER electrode were investigated. Finally, the durability of the secondary Zn–air system was assessed, demonstrating energy efficiencies of 58–61% at 20 mA cm^−2^ over 40 h in 4 M NaOH + 0.3 M ZnO at 333 K.

## Introduction

Metal-air batteries featuring oxygen electrodes are considered highly attractive systems since the electro-active material of the oxygen electrode can be easily obtained from the ambient atmosphere and does not require any storage^1^. This simplifies the design of the system whilst allowing the oxygen electrode to have infinite capacity thereby increasing the system’s energy density^2^. Metal-air batteries incorporating the use of anode materials such as Li, Al, Fe, Zn and Mg have therefore emerged as a result of their excellent specific capacities^3^. Among them, Zn–air batteries are highly capable of meeting market demands of cost, safety and environmental friendliness because Zn has several desirable properties as an anode material such as good stability in aqueous electrolytes, high specific energy, low equilibrium potential, electrochemical reversibility, good conductivity, abundance and ease of handling^4,5^. Presently, whilst primary Zn–air batteries are found in commercial applications such as hearing aids, railway signals, and navigation lights^4^, secondary Zn–air batteries possess the potential for high specific energy densities equivalent to lithium-based batteries. This makes the continued investigation of Zn–air batteries worthwhile for applications in portable electronic devices, electric vehicles, grid scale energy storage and support of renewable energy generation^6,7^.

One of the key challenges is to increase the efficiency of the oxygen reactions (i.e. oxygen reduction reaction (ORR) and oxygen evolution reaction (OER)) at the air electrode if aiming to facilitate the secondary Zn–air batteries’ commercialization. To this, effective electrocatalyst can be employed to boost reaction rate and therefore the efficiency. Currently, oxygen electrodes with bifunctional catalysts are well reported in the literature^8–10^. Although bifunctional catalysts can simplify electrode construction and reduce mass transfer losses, potentially helping to lower the production cost, in reality, a catalyst which is optimal for ORR is not usually optimal for OER and vice versa^11^. This difference in operating potentials leads to the exposure of the catalyst to wider potential ranges which may alter its surface structure over time. In addition, the interdependence of intermediate binding energies implies that active sites on the catalyst are likely to be different for each reaction which causes complications for optimisation.

**Figure. 1:**
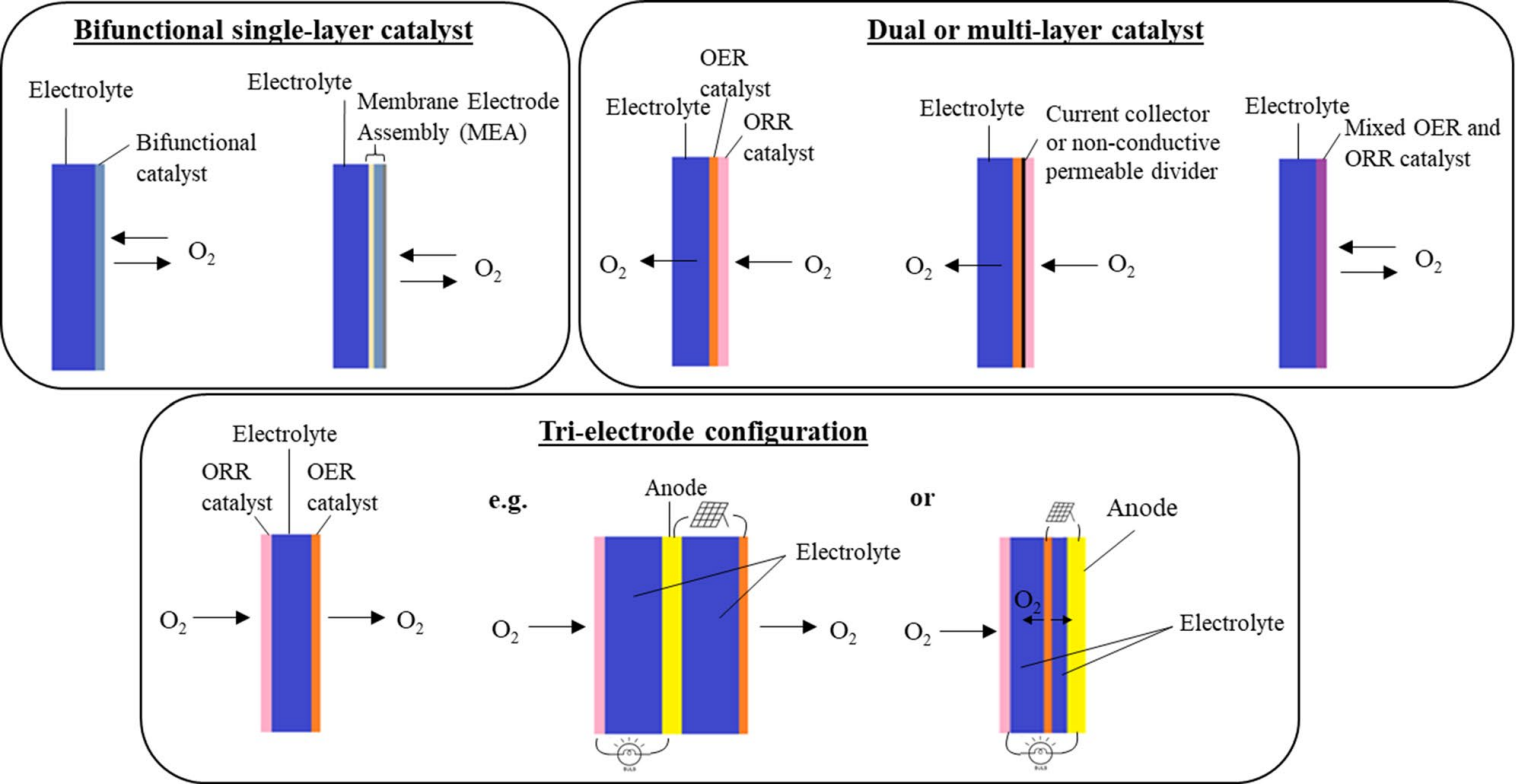
Schematic illustration of oxygen electrode designs.

Another major challenge for secondary Zn–air batteries is the construction of the oxygen electrode, largely because mono-functional catalysts for ORR and OER operate in different reaction environments. The ORR gas diffusion layer must be hydrophobic to allow gaseous oxygen to access the catalytic sites while, for OER, the electrode surface must be hydrophilic to facilitate the removal of oxygen bubbles. Figure 1 demonstrates three typical proposed secondary oxygen electrode designs which are derived from Jörissen’s review^12^, namely (i) bifunctional single-layer catalyst, (ii) dual or multi-layer catalyst and (iii) tri-electrode configuration.

For the first type of electrode design, it only involves a single layer of bifunctional catalyst which catalyses both ORR and OER, if incorporating the membrane into this design, a membrane electrode assembly (MEA) is formed as illustrated. The second type involves two (or more) layers of catalyst with different porosities and hydrophobicity to cope with the difference in reaction zones^13–15^. In some cases, the two catalyst layers are separate with the hydrophilic side for OER facing the electrolyte and semi-hydrophobic side for ORR facing the open end of the electrode^16–18^. The third electrode design involves decoupling the two reactions into two separate electrodes resulting in a tri-electrode full-cell configuration^19,20^ of two reaction-specific oxygen electrodes and a Zn electrode. Advantages and disadvantages of each of these designs are summarised in Table S1.

The implementation of electrode designs which separate the ORR and OER reaction zones have previously shown improved cycling stability^19^. This is especially true for the tri-electrode configuration, where degradation of catalysts and ancillary additives, which are not stable over the full potential range, is minimised and evolution of gas is more easily managed. For these reasons, we adopted a tri-electrode Zn–air configuration in this work.

In this paper, we initially carried out a selection of high performing ORR catalysts by comparing a number of transition metal oxides, carbonaceous materials and benchmark catalysts with rotating disc electrode (RDE) experiments. Transition metal oxides are often good electrocatalysts due to their variable oxidation states; reactions are more readily catalysed in the presence of these compounds^21^. For example, manganese oxides, cobalt oxides, and cobalt-based mixed oxides (e.g. NiCo_2_O_4_ and MnCo_2_O_4_)^22–24^ have shown promising ORR performance under alkaline conditions due to their half-filled d-orbitals, electronic energy levels for receiving electrons and increased availability of cleaving. In addition, they are more environmentally abundant and demonstrate reasonable electrical conductivity, high reaction activity and good stability. Similarly, carbonaceous materials are widely available and have the advantage of high electrical conductivity and surface area. In some cases, heteroatoms such as nitrogen, boron, phosphorus and sulphur^25^ are introduced into carbon to modify its structure and this further enhances the ORR performance of these materials.

Based on the experimental results, we incorporated selected ORR catalysts into gas diffusion electrodes (GDEs) and tested these at various current densities. The best performing ORR catalyst GDE was then assembled into our custom-built tri-electrode secondary Zn–air cell together with a reaction specific OER electrode which was optimised in our previous work^26,27^. The individual oxygen electrode potentials during consecutive discharge and charge cycling experiments were monitored to investigate the effects of operating conditions such as current density, electrolyte molarity, cell operating temperature and oxygen purity. Finally, the stability of the Zn–air secondary cell was evaluated by continuous cycling under optimal operating conditions.

## Experimental section

### Synthesis of ORR catalysts

A chemical redox method was used to prepare MnO_x_^28^—50 mL of 0.04 M KMnO_4_ (Fisher Scientific, 99%) was added into 100 mL of 0.03 M Mn(CH_3_COO)_2_ (Fisher Scientific, 98%) to form a brown precipitate. The mixture was adjusted to a pH of 12 with dilute sodium hydroxide before being centrifuged 3–5 times at 2500 rpm to collect the precipitate. The deposit was then rinsed with deionised H_2_O until the purple colour of the permanganate ions is removed. Finally, the deposit was dried overnight in air at 333 K and then ground into a powder.

The synthesis of spinel oxides Co_3_O_4_, NiCo_2_O_4_ and MnCo_2_O_4_ was carried out via a thermal decomposition method. NiCo_2_O_4_ and MnCo_2_O_4_ were prepared by adding 0.5 M (14.5 g) of nickel (II) nitrate hexahydrate, Ni(NO_3_)_2_⋅6H_2_O (Fisher Scientific, 99.9%) or 0.5 M (12.6 g) manganese (II) nitrate tetrahydrate Mn(NO_3_)_2_⋅4H_2_O (Sigma Aldrich, ≥ 97%), respectively and 1 M (29.1 g) cobalt (II) nitrate hexahydrate, Co(NO_3_)_2_⋅6H_2_O (Fisher Scientific, 98+%, ACS reagent) to methanol (Fisher Scientific, 99.9%) in a 100 mL dilution flask. The methanol was added to the transition metal nitrates in small amounts, with continuous stirring, to obtain a homogenous solution. The solution was then transferred to a crucible and heated on a hotplate leaving a dark red solid. The solid was calcined in air at 648 K for 20 h. The resulting solid was then ground down to a fine powder. The addition of Ni(NO_3_)_2_⋅6H_2_O or Mn(NO_3_)_2_⋅4H_2_O was omitted for the synthesis of Co_3_O_4_.

Graphene nanoplatelets of 300 m^2^ g^−1^ surface area (Sigma Aldrich), N-doped graphene (Sigma Aldrich), carbon black powder (Vulcan XC-72R, Cabot Corp., 100%), MnO_2_ (Sigma Aldrich) and 5 wt% Pt/C (Acros Organics) were used as received.

### Preparation of rotating disc electrode (RDE) samples

RDE (Pine Research Instrumentation) measurements were used to evaluate the activity of the various ORR catalysts in 1 M NaOH. The catalyst ink was composed of 1 mg of catalyst + 1 mL deionised (DI) H_2_O + 0.5 mL isopropanol (IPA) + 5 μL of 5wt% Nafion 117 (Sigma-Aldrich), used as received. If Vulcan XC-72R was added, the catalyst ink was composed of 0.5 mg catalyst + 0.5 mg Vulcan XC-72R + 1 mL DI H_2_O + 0.5 mL IPA + 5 μL of 5 wt% Nafion 117, to ensure the loading of material was kept consistent. The mixture was ultrasonicated for 20 min and homogenised at 28,000 rpm for 4 min with a Cole-Parmer LabGen 7 series homogeniser. After which, the ink was loaded onto the surface of a glassy carbon electrode (Pine Instrument Company) of 4 mm diameter (working area ≈ 0.126 cm^2^) in three aliquots of 8 μL with drying in between each layer to give a loading of ≈ 120 μg cm^−2^. In between uses, the glassy carbon electrode surface was consecutively wet polished with MicroCloth (Buehler) and alumina powders, 1.0 mm and 0.5 mm (MicroPolish, Buehler) then ultrasonicated in DI H_2_O.

### Preparation of electrode samples

ORR gas diffusion electrode samples were prepared following our previously described protocol^28^. Firstly, catalyst powder and Vulcan XC-72R were combined in a 1:1 weight ratio. A mixture of polytetrafluoroethylene (PTFE) solution (60 wt% in H_2_O) and solvent of 1:1 ratio IPA/H_2_O was then added to the dry powder mixture. The catalyst ink was ultrasonicated for ~ 20 min and homogenised at 28,000 rpm for ~ 4 min. The ink was then applied in thin layers onto pre-cut carbon paper (AvCarb GDS 1120) of 13 mm diameter with a spatula with drying in between until a loading of 2 mg cm^−2^ of catalyst was achieved.

OER electrodes were fabricated as reported^26,27^ by cathodic electrodeposition of Ni–Fe hydroxide based catalyst onto 15 mm by 15 mm pieces of expanded SS mesh (DeXmet Corp, 4SS 5-050). Electrodeposition was carried out in a standard three electrode half cell (~ 20 cm^3^ glass cell with polymer lid) with Pt mesh as a counter electrode and Hg/HgO in 1 M NaOH as a reference electrode. The catalyst coated SS mesh was left to dry in air before an area of ~ 0.8 cm^2^ was cut with a 10 mm carbon steel die.

Commercial ORR and OER electrodes were used as received and tested under the same conditions for comparison. The commercial ORR electrode (QSI-Nano Gas Diffusion Electrodes, Quantum Sphere, 0.35 mm thick) was composed of manganese oxide and carbon coated onto a nickel mesh current collector, whilst the commercial OER electrode (Type 1.7, Magneto special anodes, B.V.) was an expanded Ti mesh 1.3 to 1.6 mm thick, coated with Ru-Ir mixed metal oxides.

### Physical characterisation

The surface morphology and composition of the catalysts were characterized using a FEI Quanta 650 FEG scanning electron microscope (SEM) operated at high vacuum and at an accelerating voltage of 5 kV. Powder X-ray diffraction (XRD) data were collected on a Bruker D8 Advance X-ray diffractometer with Cu tube source (λ = 1.5418 Å) and analysed using Bruker Diffraction Suite EVA software.

### Electrochemical measurements

All electrochemical measurements were carried out with a Biologic SP-150 potentiostat and EC-lab software. Both RDE and GDE samples were tested in conventional three electrode set-ups consisting of a 200 cm^3^ jacketed glass cell with a Luggin capillary for the reference electrode. Pt mesh and Hg/HgO in 1 M NaOH were used as counter and reference electrodes respectively.

For RDE measurements, fresh 1 M NaOH electrolyte was used for each experiment, kept constant at 298 K with a recirculating water bath (TC120, Grant). Gaseous oxygen (BOC) was bubbled into the electrolyte through a glass frit of porosity 25–50 μm for a minimum of 30 min before each experiment. To obtain ORR polarisation curves, the potential was swept from 0.1 to − 0.5 V (*vs.* Hg/HgO) at a scan rate of 5 mV s^−1^ at a rotational speed of 400 rpm. Cyclic voltammograms were obtained by linearly scanning the potential at a rate of 50 mV s^−1^ between 0 and − 1.0 V *vs.* Hg/HgO.

For GDE measurements, the 1 M NaOH electrolyte was maintained at 333 K with a recirculating water bath. An active area of 0.8 cm^2^ was exposed to the electrolyte and a constant supply of oxygen was fed to the rear of the electrode at 200 cm^3^ min^−1^. There was a fixed distance of 10 mm between the working electrode and reference electrode and a distance of 13–15 mm between the working electrode and counter electrode. Nickel wire and mesh provided electrical contact from the gas side. Chronopotentiometric measurements were carried out at 10, 20, 50 and 100 mA cm^−2^ to evaluate the stability and performance of the electrodes.

### Zn–air tri-electrode cell

The performance of the ORR and OER electrodes was assessed in a 200 cm^3^ jacketed glass cell with PTFE insert^29^. A schematic illustration of the system is presented in Fig. S1. The electrodes in the cell were connected in a three-electrode system. The working electrode which comprised of separate reaction-specific ORR and OER electrodes, attached to a relay module (Songle, SRD-05VDC-SL-C) and microcontroller (Raspberry Pi 2014© model B+V1.2), the Zn anode as the counter electrode, and an Hg/HgO reference electrode in 4 M NaOH held at a distance of 3 mm from the Zn anode. A Python script was written to operate and control the Raspberry Pi and relay module.

The cell was modified to accommodate a Zn foil (Goodfellow, 1 mm thick, 99.95%) anode and a polymer lid enabled the electrodes to be positioned at a fixed distance of approx. 4 mm from each other. Nitrile rubber stoppers held the electrodes in position in the lid and nickel wire (Alfa Aesar, 0.5 mm dia. annealed, 99.5% Ni) was used for electrical contact of the electrodes. The Zn foil anode was initially cleaned in isopropanol followed by deionised water, then the surface of the foil was masked off with polypropylene tape (Avon, AVN9811060K, 25 μm thickness) to expose an active area of ~ 0.8 cm^2^.

All cycling experiments were carried out in an electrolyte of 4 M NaOH + 0.3 M ZnO at 333 K, unless otherwise stated. In the graphs, E_we_ vs Hg/HgO refers to the potentials of the oxygen electrodes (ORR and OER), E_ce_ vs Hg/HgO represents the potential of the Zn electrode and E_cell_ vs Hg/HgO is the full cell potential or potential difference between the half-cell potentials. Oxygen or compressed air was supplied at a constant flow rate of 200 cm^3^ min^−1^ to the back of the ORR electrode. The cycling stability and performance of the electrodes was investigated at a current density of 20 mA cm^−2^ with a cycle period of 30 min and rest period of 1 min at OCV between each half cycle. Each test was carried out for a minimum of 10 cycles and data was extracted from cycles 1, 5 and 10 to establish the state of the electrodes over time.

## Results and discussion

### Sample characterisation

**Figure 2:**
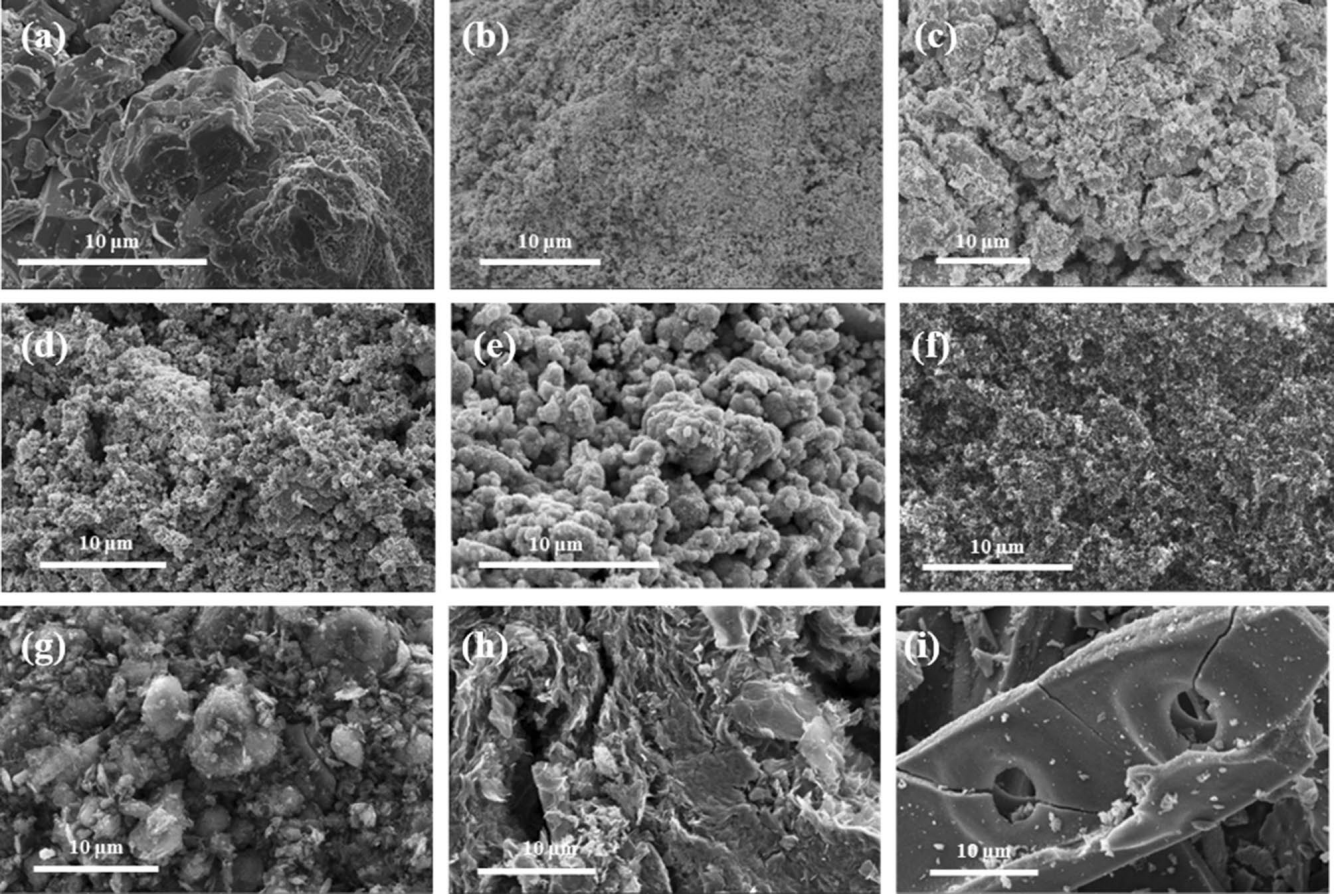
SEM images of (**a**) MnO_2_, (**b**) MnO_x_, (**c**) Co_3_O_4_, (**d**) NiCo_2_O_4_, (**e**) MnCo_2_O_4_, (**f**) Vulcan XC-72R, (**g**) graphene, (**h**) N-doped graphene and (**i**) 5 wt% Pt/C.

**Figure 3:**
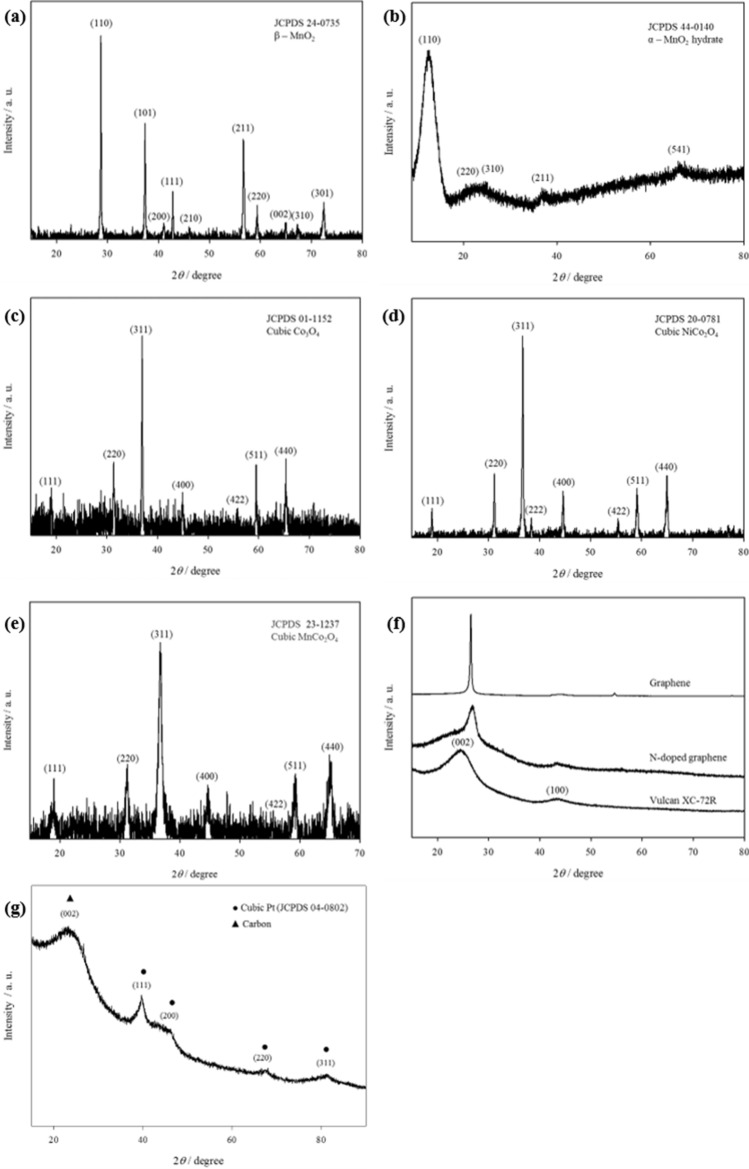
X-ray diffraction patterns of (**a**) MnO_2_, (**b**) MnO_x_, (**c**) Co_3_O_4_, (**d**) NiCo_2_O_4_, (**e**) MnCo_2_O_4_, (**f**) Vulcan XC-72R, graphene and N-doped graphene, and (**g**) 5% Pt/C.

The morphology of the ORR catalysts was characterised with SEM (Fig. 2) and X-ray powder diffraction measurements confirmed the crystal structures of the samples (Fig. 3). Structural parameters of the catalyst samples are reported in Table 1. Comparing the manganese oxides, the commercially procured MnO_2_ in Fig. 2a is composed of large granular particles and the diffraction pattern in Fig. 3a matches JCPDS 24-0735 for tetragonal β-MnO_2._ In comparison, the surface of MnO_x_ in Fig. 2b displays smaller, finer, particles and this corresponds to the diffraction pattern in Fig. 3b which exhibits weak broad peaks characteristic of a non-crystalline nature; with peaks at 13.3°, 25°, 37° and 66° matching the (110), (220), (310), (211) and (541) peaks of tetragonal body-centred α-MnO_2_ hydrate, JCPDS 44-0140^28^.

**Table 1:** Summary of XRD structures from Fig. 2

Catalyst	Crystal system	Space group	Lattice constant/Å	Volume of cell/Å^3^
MnO_2_	Tetragonal	P42/mnm	a = b = 4.4 c = 2.87	55.6
MnO_x_	Amorphous	n/a	n/a	n/a
Co_3_O_4_	Cubic	Fd3m	a = b = c = 8.09	529.5
NiCo_2_O_4_	Cubic	Fd3m	a = b = c = 8.11	533.4
MnCo_2_O_4_	Cubic	Fd3m	a = b = c = 8.28	567.7
XC-72R	Hexagonal	P 63/m m c	a = b = 2.41 c = 7.26	42.2
Graphene	Hexagonal	P 63/m m c	a = b = 2.38 c = 6.72	38.1
N-doped graphene	Hexagonal	P 63/m m c	a = b = 2.41 c = 6.68	38.8
5 wt% Pt/C	Cubic Pt	Fm3m	a = b = c = 3.92	60.2

In Fig. 2c–e, the surface morphologies of cobalt based oxides Co_3_O_4_, NiCo_2_O_4_ and MnCo_2_O_4_ comprise particle clusters of irregular sizes. These transition metal oxides were all found to have spinel structures with similar cubic crystal systems in Fig. 3c–e (JCPDS 01-1152, JCPDS 20-0781 and JCPDS 23-1237, respectively). This demonstrates the ability of the thermal decomposition method to yield highly crystalline metal oxides, as supported by the intense, clearly defined peaks in the diffraction patterns.

The SEM images of the carbon materials show greater variation. In Fig. 2f, Vulcan XC-72R carbon black is composed of closely packed nanoparticles. In contrast, the appearance of graphene in Fig. 2g is that of highly disordered platelets with some agglomeration. The N-doped graphene (Fig. 2h) however, appears to be constructed of thin-layered sheets. Corresponding diffraction patterns of Vulcan XC-72R, commercial graphene nanoplatelets and N-doped graphene in Fig. 3f all display the (002) and (100) peaks of carbon at slightly varying 2*θ* values. Vulcan XC-72R is identified as hexagonal graphite from JCPDS 41-1487 with (002) and (100) peaks present at 24.5° and 43.2° respectively. Likewise, the (002) and (100) peaks of N-doped graphene are present at 26.7° and 43.3°, respectively. The background intensity observed in the diffraction patterns of both Vulcan XC-72R and N-doped graphene is attributed to the highly disordered nature of these materials as seen in their surface morphology. In contrast, the diffraction pattern of graphene nanoplatelets displays a sharp, intense (002) peak at 26.5° and a small broad (100) peak at 44° which indicates the more crystalline nature of this sample.

Lastly, in Fig. 2i, SEM images of 5 wt% Pt/C present rod-like fragments of carbon with circular voids. Cubic Pt is identified from the majority of the peaks in the diffraction pattern of 5 wt% Pt/C in Fig. 3g and the peak at 23° is attributed to the (002) peak of carbon present.

### Rotating disc electrode (RDE) measurements

**Figure 4:**
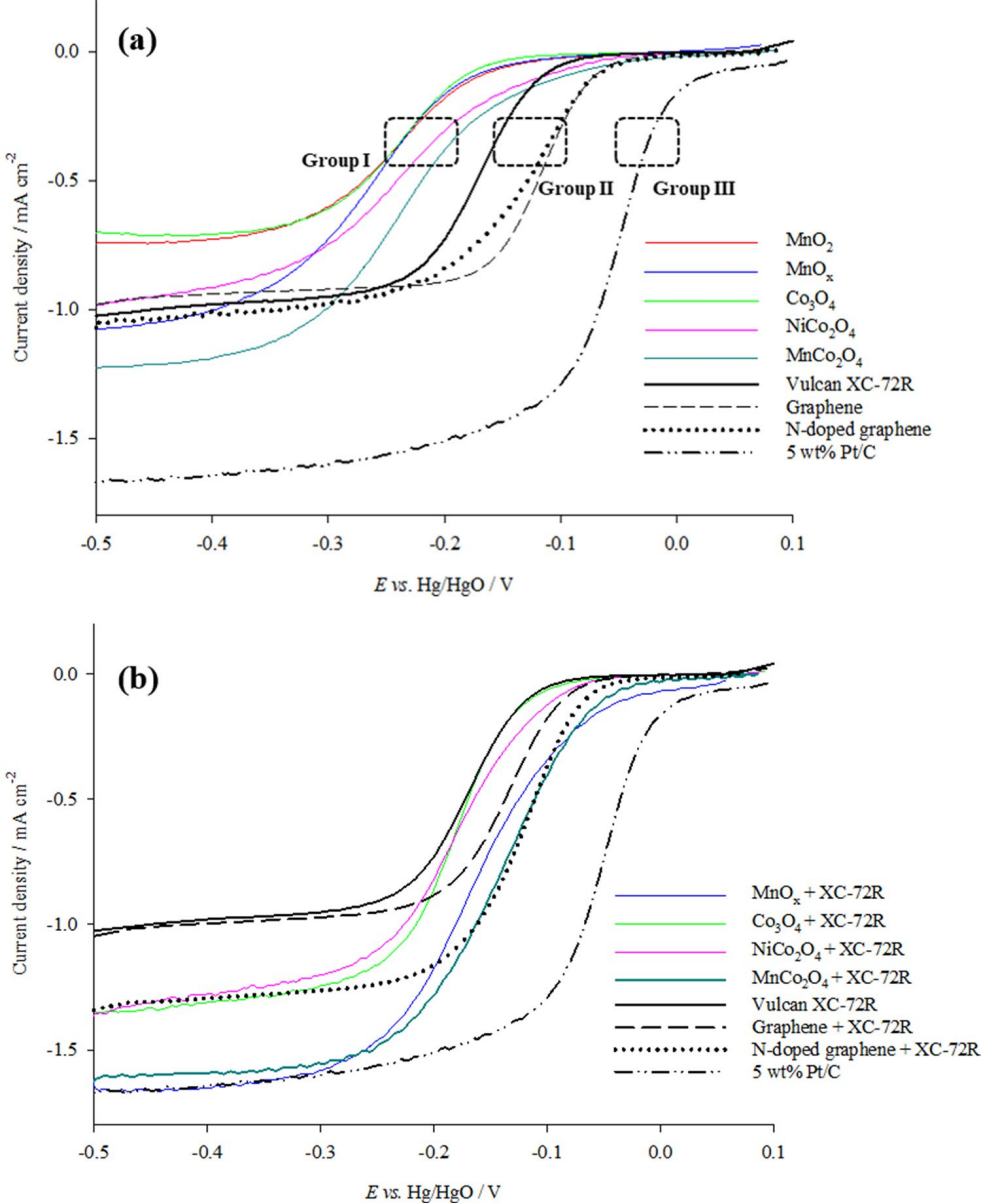
Linear sweep voltammograms of thin films of (**a**) catalysts and (**b**) catalysts with XC-72R, measured on glassy carbon RDE tip at 400 rpm, scan rate of 5 mV s^−1^ in O_2_ saturated 1 M NaOH at 298 K.

Linear sweep voltammograms were recorded for the ORR catalysts at a scan rate of 5 mV s^−1^. The plots collected (Fig. 4a) are generally sigmoidal in shape extending into plateaus at more negative potentials due to mass transfer limitation. The limiting current densities, *j*_*L*_, *E*_*1/2*_ potentials (at which *j*/*j*_*L*_ = ½) and onset potentials at − 0.1 mA cm^−2^ were extracted from these plots and presented in Table 2. Notably, in Fig. 4a the catalysts are able to be grouped according to their *E*_*1/2*_ potentials as: (I) metal oxides, (II) carbon materials and, (III) precious metals.

**Table 2:** ORR kinetic parameters from LSV, Tafel and K-L plots of catalysts.

Catalyst	$E_{{ - 0.1\;{\text{mA}}\;{\text{cm}}^{{ - 2}} }}$ *vs.* Hg/HgO/V	*E*_*½*_* vs.* Hg/HgO/V	*j*_*L*at 0.4 V_/mA cm^−2^	Tafel slope/mV dec^−1^	Slope, K/mA^−1^ cm^2^ rpm^−1/2^	Intercept, *j*_k_/mA^−1^ cm^2^	Apparent electron transfer number, n_app_
MnO_2_	− 0.174	− 0.236	− 0.73	− 115	− 16.3	− 0.56	2.6
MnO_x_	− 0.177	− 0.261	− 1.01	− 110.1	− 9.1	− 0.57	4.6
Co_3_O_4_	− 0.186	− 0.236	− 0.71	− 119.5	− 19.1	− 0.48	2.2
NiCo_2_O_4_	− 0.119	− 0.229	− 0.92	− 169.4	− 13.6	− 0.45	3.1
MnCo_2_O_4_	− 0.102	− 0.228	− 1.19	− 168	− 9.9	− 0.36	4.2
XC-72R	− 0.117	− 0.173	− 0.98	− 70.3	− 18.8	− 0.12	2.2
Graphene	− 0.073	− 0.116	− 0.95	− 60.2	− 19.2	− 0.13	2.2
N-doped graphene	− 0.074	− 0.129	− 1.01	− 67.5	− 15.3	− 0.20	2.7
5 wt% Pt/C	+ 0.020	− 0.055	− 1.65	− 63.5	− 10.3	− 0.11	4.0

Single metal oxides of Mn and Co in Group I display onset potentials at − 0.17 V and − 0.19 V respectively, as well as *E*_*1/2*_ values between − 0.24 and − 0.26 V. The reduction reactions of these metal oxides, in Eqs. (1) and (2), occur around the onset potentials in Fig. 4a and coincide with the standard potential of the first 2e^−^ step of the indirect pathway of ORR in Eq. (3).

1 $${\text{Co}}_{{3}} {\text{O}}_{{4}} + {\text{ 4H}}_{{2}} {\text{O }} + {\text{ 2e}}^{ - } \to {\text{3Co}}\left( {{\text{OH}}} \right)_{{2}} + {\text{ 2OH}}^{ - } \quad {\text{E}}^{\uptheta } = \,- 0.{\text{19 V}}\;vs.\;{\text{Hg}}/{\text{HgO}}$$

2 $${\text{MnO}}_{{2}} + {\text{ H}}_{{2}} {\text{O }} + {\text{ e}}^{ - } \to {\text{MnOOH }} + {\text{ OH}}^{ - } \quad {\text{E}}^{\uptheta } = \,- 0.{\text{15 V}}\;vs.\;{\text{Hg}}/{\text{HgO}}$$

3 $${\text{O}}_{{2}} + {\text{ H}}_{{2}} {\text{O }} + {\text{ 2e}}^{ - } \to {\text{HO}}_{{2}}{^{ - }} + {\text{ OH}}^{ - } \quad {\text{E}}^{\uptheta } = \,- 0.{\text{165 V}}\;vs.\;{\text{Hg}}/{\text{HgO}}$$

Mixed metal oxides of MnCo_2_O_4_ and NiCo_2_O_4_ in the same group display slightly more positive onset potentials at − 0.10 and − 0.12 V respectively but retain *E*_*1/2*_ values approx. − 0.23 V.

Group II carbon materials display more positive *E*_*1/2*_ values than the metal oxides in Group I. The graphene materials have onset potentials at − 0.07 V and *E*_*1/2*_ values of − 0.11 V whilst the onset potential and *E*_*1/2*_ for Vulcan XC-72R are at − 0.12 V and − 0.17 V respectively. In Group III, 5 wt% Pt/C presents the most positive onset potential at 0.02 V, an *E*_*1/2*_ of − 0.055 V and greatest limiting current density at − 0.4 V as oxygen reduction proceeds via the 4e^−^ pathway^30^. Pt/C also has the smallest *E*_*1/2*_ value due to its high electrical conductivity and reversible kinetics for the ORR reaction.

Tafel slope analysis of the various catalysts is given in Fig. S2a. The kinetically controlled region for 5 wt% Pt/C commences at 0.02 V *vs.* Hg/HgO whilst the kinetically controlled region of the metal oxides and carbon materials occur at more negative potentials in the range of − 0.03 to − 0.1 V. The Tafel slope value of Pt/C is − 63.5 mV dec^−1^, typical of Pt at low current density dE/d log i = − 2.3 RT/F^31,32^ and characteristic of a reaction pathway where the rate determining step involves a transition of oxygen from physisorption to chemisorption^33,34^. Tafel slope values of the carbon materials are in the same region (− 60 to − 70 mV dec^−1^) as Pt/C indicating that these materials have a similar ORR pathway. Single metal oxides of Co and Mn report Tafel slope values in the region of − 110 to − 120 mV dec^−1^ which is representative of dE/d log i = − 2.3 2RT/F, where the rate determining step is the first electron transfer step^35,36^. Both NiCo_2_O_4_ and MnCo_2_O_4_ mixed metal oxides report slightly higher slope values around − 170 mV dec^−1^ indicative of the presence of OH^−^ ions and H_2_O at the surface of the oxide which hinders the oxygen adsorption and electron transfer thus affecting the oxygen reduction pathway^35^.

The Koutecky-Levich (K-L) equation was used to determine the reaction kinetic parameters for the various catalyst samples without mass transfer contribution. In Eq. (4), the total measured current density, j, is the sum of the current density from electron transfer and mass transfer.

4 $$\frac{1}{j}=\frac{1}{{j}_{k}}+\frac{1}{{j}_{L}}$$

*j*, *j*_*k*_ and *j*_*L*_ are the total measured, kinetic and limiting current density respectively.

5 $${j}_{L}=0.201 \;nFA{D}^\frac{2}{3}{\upsilon }^{-\frac{1}{6}}C{\omega }^\frac{1}{2}$$

From Eq. (5), the limiting current density, j_L_ is proportional to the square root of rotation speed. As such, the K-L Eq. (6) describes a linear plot of *j*^*−1*^* vs. ω*^*−1//2*^, where the intercept is j_k_ and the slope of the plot is K.

6 $$\frac{1}{j}=\frac{1}{{j}_{k}}+\frac{1}{K{\omega }^\frac{1}{2}}$$

7 $$\mathrm{Hence}, \quad K=\frac{{\upupsilon }^\frac{1}{6}}{0.201 \; nF{D}^\frac{2}{3}C}$$

where *υ* is the kinematic viscosity of electrolyte 1 M NaOH (1.1 × 10^−2^ cm^2^ s^−1^)^37^, D is the diffusion coefficient of O_2_ in 1 M NaOH (1.89 × 10^–5^ cm^2^ s^−1^)^38^, *ω* is the rotational speed in rpm and C is the concentration of oxygen in the bulk solution (8.4 × 10^–7^ mol cm^−3^)^38^.

Linear sweep voltammograms were collected with the RDE at rotation rates of 100, 400, 900, 1600 and 2500 rpm. Values were taken from − 0.4 V in the mass transfer limited region to produce K-L plots i.e. *–j*^*−1*^* vs. ω*^*−1//2*^ for the catalysts (Fig. S3a). Using Eqs. (6) and (7), indicators of catalyst performance such as the kinetic current density without mass-transfer effects, j_k_, were determined from the y-intercept, and the electron transfer number, from the gradient of the plot, *K*. These are presented in Table 2.

5 wt% Pt/C and XC-72R have the smallest absolute *j*_*k*_ values signifying faster kinetics for these materials. The slope of the plot for XC-72R however, is almost double that of 5 wt% Pt/C which is expected as *K* is an indicator of the electron transfer number during oxygen reduction reaction. Theoretically the K-L plot for 5 wt% Pt/C should pass through the origin under mass transport limiting conditions^39^ however this was not observed in Fig. S3a suggesting that there were kinetic or diffusional limitations which affected the results. This could be due to a slight inconsistency in topology and morphology of the Pt/C catalyst film which was shown by Garsany et al*.*^40^ to affect the accuracy of ORR activity values. Since all catalyst films were prepared in the same manner however, any effect on the results should be similar for all samples. The intercept of the K-L plot of graphene, ≈ − 0.13 mA^−1^ cm^2^, is comparable to that for XC-72R, however the intercept of the K-L plot of N-doped graphene, − 0.20 mA^−1^ cm^2^, indicates that current density is more dependent on voltage at this catalyst. This is likely to be due to the doping of graphene with nitrogen which reduces overall conductivity, contributing to slower electron transfer kinetics. Conversely, the absolute *K* value for N-doped graphene is smaller than that for graphene as the presence of nitrogen helps to create more active sites for ORR^41,42^.

The intercepts of largest absolute value, − 0.57 mA^−1^ cm^2^, were observed for the Mn-based oxides. Despite this, the absolute *K* value for MnO_x_ is much lower than that of MnO_2_, close to the *K* value for 5 wt% Pt/C. The electron transfer number is determined to be approx. 4 for MnO_x_ but close to 2 for MnO_2._ This is consistent with results in the literature which report an electron transfer number of 4 in the ORR pathway of α-MnO_2_ and a tendency for the electron transfer number to be less than 4 for β-MnO_2_^43^. The ORR pathway therefore varies for different polymorphic forms of Mn-oxide based catalyst although the rate of the chemical step remains approximately the same. The electron transfer number particularly for the MnO_x_ and MnCo_2_O_4_ catalysts slightly exceeds 4, as the reduction of the manganese oxides present in these catalysts occur in parallel with oxygen reduction. In previous work, we showed that in N_2_ saturated solution the electrochemical reduction of manganese oxide takes place in the same potential range as oxygen reduction^28^. This contribution from side reactions results in the calculated electron number being slightly greater than 4.

The intercept for Co_3_O_4_, ≈ − 0.48 mA^−1^ cm^2^, is less negative compared to both forms of Mn-oxide and the apparent electron transfer number is determined to be 2 from the *K* value_._ Substituting Co for Ni in NiCo_2_O_4_ and Mn in MnCo_2_O_4_ results in a reduction of the absolute *K* value, signifying an improvement in electron transfer kinetics in mixed metal oxides.

### Significance of carbon in the ORR catalyst layer

A carbon support material is incorporated into the ORR catalyst ink to enhance conductivity and assist in forming a suitable three-phase boundary in the gas diffusion electrode. Vulcan-XC-72R was selected as it is inexpensive, has a high surface area of 250 m^2^ g^−1^ and low electrical resistivity of 0.08 to 1 Ω cm^44,45^. LSV plots of catalyst samples mixed with Vulcan XC-72R at 400 rpm are shown in Fig. 4b. The most visible effect of the addition of Vulcan XC-72R was an increase in limiting current density. This was noted to be more evident for metal oxides with an additional 0.60 mA cm^−2^ for single metal oxides and 0.40 mA cm^−2^ for mixed metal oxides, whilst a 0.28 mA cm^−2^ increase was observed for N-doped graphene and a 0.05 mA cm^−2^ increase for graphene. The inclusion of Vulcan XC-72R in the catalyst ink also resulted in positive shifts of onset and half wave potentials, *E*_*1/2*_ for all catalysts with the exception of graphene. These changes are a probable outcome of the increase in utilisation of electrochemical surface area^46^ and improvement in contact between catalyst particles on Vulcan XC-72R supported catalysts^47^.

Corresponding Tafel plots and kinetic parameters for these catalyst mixtures are presented in Fig. S2b and Table 3 respectively. Tafel slope values of MnO_x_ and graphene materials with and without XC-72R were similar, indicating that their ORR reaction pathways were not affected. Co-based oxides—Co_3_O_4_, NiCo_2_O_4_ and MnCo_2_O_4_ however, produced less negative Tafel slope values between − 68 and − 80 mV dec^−1^ when combined with XC-72R, denoting a change in the ORR pathway. K-L plots for the catalyst samples combined with Vulcan XC-72R are given in Fig. S3b. In general, a decrease in absolute *j*_*k*_ values was observed for all catalysts mixed with XC-72R. MnO_x_ exhibited the largest decrease in absolute *j*_*k*_ value by 55 mA^−1^ cm^2^_,_ whilst NiCo_2_O_4_ recorded a 32 mA^−1^ cm^−2^ decrease and graphene displayed the smallest decrease of 5 mA^−1^ cm^2^. It can be deduced that the impact of Vulcan XC-72R on catalyst performance is subject to the initial ORR activity of the catalyst.

**Table 3:** ORR kinetic parameters from LSV, Tafel and K-L plots of catalysts combined with XC-72R.

Catalyst	$E_{{ - 0.1\;{\text{mA}}\;{\text{cm}}^{{ - 2}} }}$ *vs.* Hg/HgO/V	*E*_*½*_* vs.* Hg/HgO/V	*j*_*L*at 0.4 V_/mA cm^−2^	Tafel slope/mV dec^−1^	Slope, K/mA^−1^ cm^2^ rpm^−1/2^	Intercept, *j*_k_/mA^−1^ cm^2^	Apparent electron transfer number, n_app_
MnO_x_ + XC-72R	− 0.029	− 0.163	− 1.65	− 130.8	− 12.8	− 0.02	3.3
Co_3_O_4_ + XC-72R	− 0.114	− 0.183	− 1.32	− 74.6	− 11.5	− 0.18	3.6
NiCo_2_O_4_ + XC-72R	− 0.093	− 0.180	− 1.28	− 68.2	− 13.1	− 0.13	3.2
MnCo_2_O_4_ + XC-72R	− 0.049	− 0.141	− 1.59	− 80.8	− 10.7	− 0.11	3.9
Graphene + XC-72R	− 0.086	− 0.137	− 1.00	− 57.3	− 16.6	− 0.18	2.5
N-doped graphene + XC-72R	− 0.066	− 0.122	− 1.29	− 65.9	− 15.1	− 0.06	2.8

The *K* values for NiCo_2_O_4_, MnCo_2_O_4_, graphene and N-doped graphene are not significantly affected by the presence of Vulcan XC-72R. However, the *K* value for Co_3_O_4_ decreased considerably with the addition of Vulcan XC-72R, representing an increase in electron transfer number for the ORR pathway. This synergetic coupling of Co_3_O_4_ with a carbon component is reported in literature^48,49^. Without a carbon support, Co_3_O_4_ is proposed to favour the disproportionation of HO_2_^−^ into O_2_ and OH^−^^50,51^ which agrees well with the electron transfer number of approximately 2 for Co_3_O_4_ in Table 2. Therefore, it is expected that the physical adsorption of Co_3_O_4_ onto a carbon support yields a 2 + 2, four electron ORR pathway^52^ that begins with the electroreduction of O_2_ to HO_2_^−^ (Eq. 1) at the Co_3_O_4_ catalyst and Vulcan XC-72R interface followed by rapid disproportionation of the HO_2_^−^ at the metal oxide surface into O_2_ which is then further electro-reduced.

In contrast, the absolute *K* value for MnO_x_ increased with the addition of Vulcan XC-72R, which is representative of a decrease in the electron transfer number from 4.6 to 3.3 (Table 3). This is attributed to the presence of two sites on the carbon-catalyst composite for the two step electron pathway. The initial reduction of O_2_ into HO_2_^−^ takes place at the carbon support more readily, causing a small increase in preference for the two electron ORR pathway^53^.

**Figure 5:**
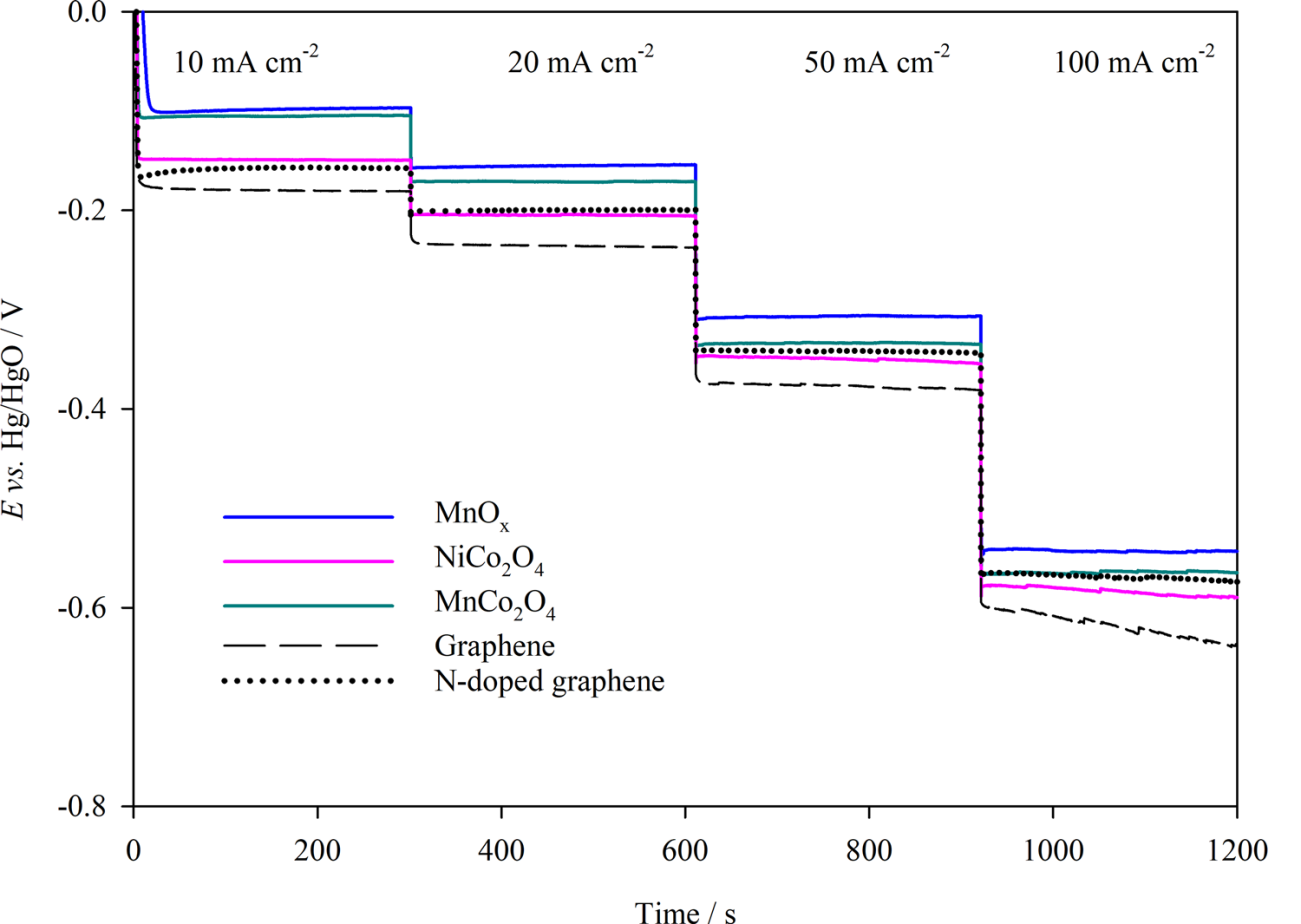
Chronopotentiometric measurements of GDE samples at 10 to 100 mA cm^−2^ in 1 M NaOH, 333 K, O_2_ flow rate of 200 cm^3^ min^−1^.

The stability of the catalysts was evaluated in a GDE half-cell over a range of current densities. Figure 5 gives the potential versus time plots for GDEs of MnO_x_, MnCo_2_O_4_, NiCo_2_O_4_, graphene and N-doped graphene. MnO_x_ showed good overall stability and performance for ORR at low and high current densities signifying its suitability for further optimisation.

MnCo_2_O_4_ also appears to retain good stability for ORR over the range of current densities however at higher current densities of 50 and 100 mA cm^−2^, larger overpotentials are observed indicating that MnCo_2_O_4_ does not perform as well as MnO_x_. The GDE of graphene displayed the lowest ORR performance over the range of current densities tested, exhibiting a rapid deterioration in performance at 100 mA cm^−2^_._ Therefore, under the selected experimental conditions, MnO_x_ GDE was chosen for further tests in the secondary Zn–air system.

### Zn–air tri-electrode cell performance

#### Electrode selection

The performance of MnO_x_ on C paper was further optimised^28^ and evaluated against MnCo_2_O_4_ on C paper and a commercial ORR electrode, QSI-Nano (Fig. S4). Across cycles 1 to 10, the MnCo_2_O_4_ electrode exhibited poor performance with overpotentials of 65 mV greater than that of the commercial ORR electrode and MnO_x_ electrode. The initial performance of the commercial ORR and MnO_x_ catalyst coated electrodes appears similar, however the overpotential of the QSI-Nano electrode increased at a constant rate over 10 cycles due to electrode degradation. In addition, the mass of the commercial electrode is nearly four times that of the MnO_x_ catalyst coated electrode, indicating that the MnO_x_ electrode is not only more stable over time but also has greater activity per mass.

**Figure 6:**
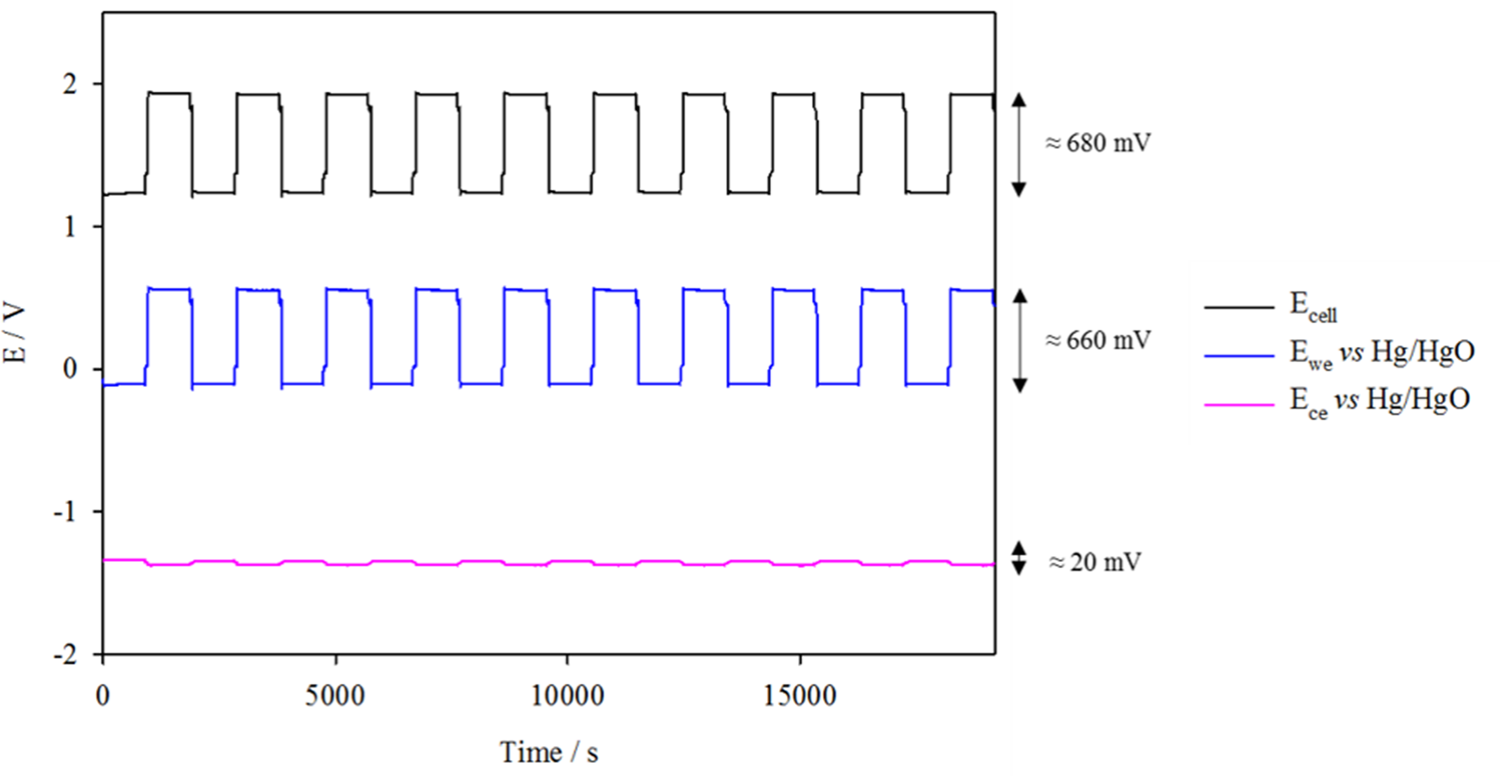
Galvanostatic cycling data at 20 mA cm^−2^ with 30 min cycle periods and 1 min OCV after each charge/discharge step. Oxygen electrodes MnO_x_ on C paper (ORR) and Ni-Fe-Co(OH)_2_ on SS mesh (OER) were positioned on either side of the Zn anode in static 4 M NaOH + 0.3 M ZnO at 333 K and O_2_ flow rate of 200 cm^3^ min^−1^.

Figure 6 displays the consecutive charge and discharge cycling data at 20 mA cm^−2^ of the tri-electrode system with MnO_x_ on C paper (ORR) and Ni-Fe-Co(OH)_2_ on SS mesh (OER) oxygen electrodes. As labelled in the figure, the polarisation of E_we_ (oxygen electrode) was 660 mV whilst polarisation of E_ce_, (zinc electrode) was only 20 mV, indicating that there is a distinctly greater contribution from the oxygen electrode towards the overall overpotential. The voltage efficiency of the system was calculated to be approx. 64%. Since Zn foil was used at the anode, the coulombic efficiency of the system is 100%, and energy efficiency has the same value as the voltage efficiency—64%. This demonstrates the strong connection between the oxygen electrode overpotentials and energy efficiency of the system.

### Effect of operating conditions

#### Current density

**Figure 7:**
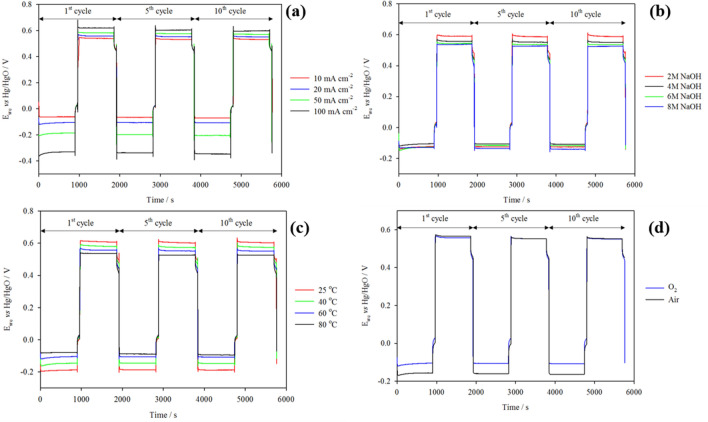
Effects of (**a**) current density, (**b**) electrolyte molarity, (**c**) temperature and, (**d**) O_2_ purity on oxygen electrode performance in static electrolyte 4 M NaOH + 0.3 M ZnO at 333 K, O_2_ or compressed air was supplied at 200 cm^3^ min^−1^.

The performance of the oxygen electrodes was monitored at current densities 10 mA cm^−2^, 20 mA cm^−2^, 50 mA cm^−2^ and 100 mA cm^−2^ as shown in Fig. 7a. At higher current densities, larger overpotentials were observed at both oxygen electrodes although the increases in overpotential at the ORR electrode were three to five times greater than at the OER electrode. In Table S2, the ORR electrode potential initially rose by ~ 40 mV between 10 to 20 mA cm^−2^. Subsequent tests at 20 mA cm^−2^ to 100 mA cm^−2^ showed increases in overpotential of around 30 mV per 10 mA cm^−2^ signifying a strong dependence of the ORR performance on current density. Similarly, the OER electrode potential was observed to increase at a decreasing rate with current density. OER electrode potential at 20 mA cm^−2^ was 20 mV higher than at 10 mA cm^−2^. However the overpotential only rose by 20 mV between 20 and 50 mA cm^−2^ to 0.571 V, and to 0.596 V at 100 mA cm^−2^. Since increases in overpotential at the OER electrode were not directly proportional to increases in current density, this implies that the OER performance is much less dependent on this parameter. The increases in ORR overpotential with current density however are greater as it is likely the rate of ORR depends on factors such as mass transfer limitations within the catalyst layer.

On the contrary, the potential window between the Zn electrode charge and discharge potentials increased consistently with current density (approx. 10 mV every 10 mA cm^−2^) indicating that the Zn electrode performance has a strong dependence on the operating current. In a static 0.3 M ZnO-based electrolyte at current densities up to 100 mA cm^−2^, mass transport becomes significant due to increasing concentration polarisation. During charge, the transport of Zn(OH)_4_^2−^ to the electrode will be limited, whilst during discharge a passivation layer of Zn(OH)_2_ may hinder zinc oxidation. The contribution of the Zn overpotential to the overall overpotential of the system over the range of current densities tested, however, is still significantly lower than that from the oxygen electrodes. The results report a decrease in overall voltage and energy efficiency of 18% going from 10 to 100 mA cm^−2^.

#### Electrolyte molarity

The molarity of the NaOH electrolyte was varied from 2 to 8 M whilst ZnO concentration was kept at 0.3 M. The conductivities of these electrolytes were measured with a Jenway, 4310 benchtop conductivity meter at 333 K and presented in Table S6. The measured conductivity values were not observed to increase linearly with molarity which is in agreement with previous work (Fig. S6). As the concentration increased from 2 to 4 M NaOH, an increase of 89 mS cm^−1^ was recorded, whereas between 4 and 6 M NaOH, the conductivity only rose by 9 mS cm^−1^ and an increase of just 7 mS^−1^ was seen as the concentration increased from 6 to 8 M.

From the experimental results, the NaOH molarity has different effects on the oxygen and Zn electrodes. In Fig. S5, the oxidation and reduction potentials of the Zn anode shift to more negative values with increasing electrolyte concentration, whilst retaining the same potential separation of ~ 30 mV. The Zn anode half reaction is given as

8 $${\text{Charge}}{:} \; {\text{Zn}}\left( {{\text{OH}}} \right)_{{4}}{^{{{2} - }}} + {\text{ 2e}}^{ - } \leftrightarrow {\text{ Zn }} + {\text{ 4OH}}^{ - } \quad {\text{E}}^{\uptheta } = \, - {1}.{\text{25 V}}\;vs.\;{\text{SHE}}$$

The overall cathodic reaction, as proposed by Bockris^54^, involves the reduction of Zn^2+^ to Zn^+^ and then Zn, through two electron transfer steps and the rate determining step is the same regardless of the direction in which the reaction proceeds. The gradual shift in observed potentials towards more negative values for the charge and discharge reaction of the Zn anode agrees well with observations by Bockris of the Zn rest potential, which is affected by the pH of the alkaline zincate solution.

The response of the ORR electrode is given in Fig. 7b. It is noted that in cycle 1, the ORR potentials in 6 M and 8 M NaOH electrolyte are slightly greater, this being due to the higher viscosities of the electrolyte at these concentrations (Fig. S6). The system is presumed to have equilibrated by cycle 5 as the ORR potentials remain consistent up to cycle 10. The ORR electrode performance in 2 M NaOH + 0.3 M ZnO displayed the highest ORR potential due to the low conductivity of the reaction environment. At 4 M NaOH + 0.3 ZnO, the ORR potential was reduced significantly by ~ 20 mV, which can be attributed to the increase in conductivity. However, subsequent increases in NaOH molarity to 6 M and 8 M resulted in higher ORR potentials. This is because at ≥ 6 M, the increase in conductivity is limited by the increasing viscosity of the electrolyte (Fig. S6) and decreasing oxygen solubility and diffusivity^55^, both of which contribute to a lower concentration of oxygen at the three phase boundary. Additionally, since the ORR results in the production of OH^–^ ions, a high concentration of OH^–^ in the electrolyte can hinder the rate of protonation of O_2_^–^ to HO_2_^–^ thereby increasing the reaction overpotential. In contrast, the OER electrode performance was observed to improve with increasing electrolyte molarity. This improvement can be related to the changes in adsorption energy of intermediates in the OER reaction pathway which is influenced by pH^56,57^. In an electrolyte molarity of 4 M, the potential of the OER electrode was ~ 30 mV less than that at 2 M. The OER electrode potential was further reduced in 6 M NaOH by ~ 20 mV and by another 10 mV in 8 M.

It should be noted that the polarisation, ΔE, between ORR and OER was comparable in 4 M and 6 M NaOH electrolyte as the improvement in OER performance offsets the reduction in ORR performance. Voltage and energy efficiencies of the system in both 4 M and 6 M electrolyte concentration were found to be similar ~ 64–65% (Table S3). As such, 4 M NaOH was chosen for further tests to avoid issues associated with higher viscosities.

#### Temperature

The temperature of the electrolyte was varied between 25 and 80 °C. A lower limit of 25 °C was selected to mimic room temperature conditions and an upper limit of 80 °C was chosen as temperatures higher than this would result in rapid loss of water from the electrolyte through evaporation. From the plots in Fig. 7c, it is clear that as temperature increases the overpotentials of both oxygen reactions decrease.

Based on values in Table S4, the reduction in the potential window between ORR and OER occurs at a decreasing rate with increases in temperature. The polarisation between 25 and 40 °C initially decreased by ~ 5 mV °C^−1^. Subsequently between 40 and 60 °C, the polarisation was reduced by ~ 3 mV °C^−1^, and increasing the temperature from 60 to 80 °C resulted in a ~ 2 mV °C^−1^ reduction in polarisation. Taking a closer look at the individual oxygen electrodes, the effect of temperature is initially more significant at the ORR electrode than at the OER electrode, particularly between 25 and 40 °C where a ~ 3 mV °C^−1^ decrease in ORR overpotential is observed in comparison to a ~ 2 mV °C^−1^ reduction in overpotential at the OER electrode. However between 60 and 80 °C, the effect of temperature becomes more comparable with a ~ 1 mV °C^−1^ reduction in overpotential at both electrodes.

Equally, the potential window between the Zn electrode reactions was greater at lower temperatures, decreasing by 0.6 mV °C^−1^ between 25 and 40 °C. Subsequently between higher temperatures of 60 °C and 80 °C, the potential gap was reduced by only ~ 0.2 mV °C^−1^ indicating that the Zn electrode reactions are much less sensitive to changes in temperature compared to the oxygen electrode reactions. Since voltage and energy efficiencies are mainly influenced by the potential window between ORR and OER, efficiency values are observed to increase at a decreasing rate accordingly with rises in temperature.

#### Compressed air vs. bottled O_2_

As air is typically composed of 21% oxygen, the use of compressed air in the system results in a 3% reduction in energy and voltage efficiency (Fig. 7d and Table S5) due to a 56 mV increase in overpotential at the ORR electrode. The performance of both OER and Zn electrodes remain unaffected by the use of compressed air over bottled oxygen.

Overall changes in overpotential at the ORR and OER electrodes were plotted against these operating parameters in Fig. S7a–c. The effect of compressed air versus bottled O_2_ was omitted as it is clear that this parameter only affects the ORR electrode. The ORR electrode performance appears to be more sensitive to changes in temperature and current density which suggests that a clear current path is vital for ensuring good ORR performance hence the catalyst layer and electrode structure are of particular importance for the ORR electrode. The transport of reactants to the three-phase boundary within the ORR electrode catalyst layer is equally important as electrolyte temperature, which alters the electrolyte viscosity and solubility of oxygen in the electrolyte, producing a more noticeable effect on the rate of change of ORR overpotential as well. In contrast, the OER electrode is more greatly influenced by electrolyte molarity than the ORR electrode, likely because OER requires OH^−^ ions for the evolution of O_2_. Thus, a greater concentration of OH^–^ ions in the reaction environment brings about a greater decrease in overpotential at the OER electrode.

### Durability tests

**Figure 8:**
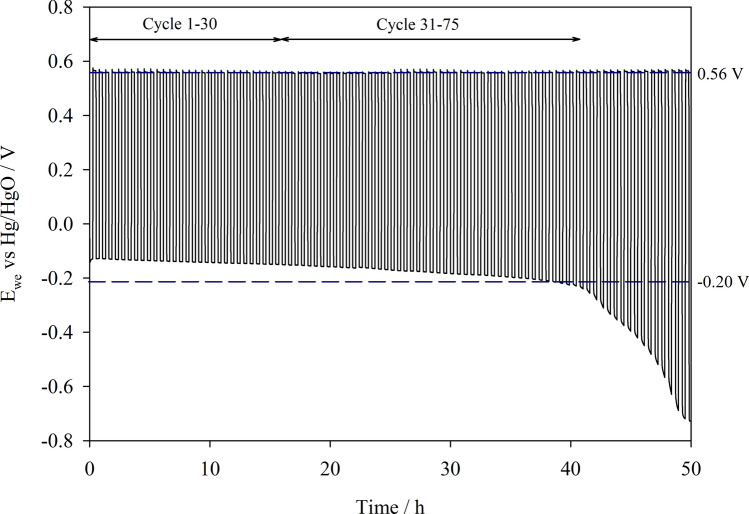
Galvanostatic cycling performance of oxygen electrodes MnO_x_ + Ni-Fe-Co(OH)_2_ in a tri-electrode Zn–air set-up over 50 h at 20 mA cm^−2^ with 30 min cycle periods, in static electrolyte 4 M NaOH + 0.3 M ZnO at 333 K. Compressed air was supplied at 200 cm^3^ min^−1^.

The overall performance and stability of the oxygen electrodes was assessed over a longer period of time by cycling at 20 mA cm^−2^ for 50 h in compressed air. The OER electrode was placed between the Zn electrode and ORR electrode for these cycling tests as this configuration is more practical for applications since the same face of the Zn electrode is used for both discharge and charge reactions. The growth of Zn dendrites over the extended cycling period is also better managed in this configuration, preventing premature short circuits. Results in Fig. 8 show that the oxygen potentials are not significantly affected by the electrode arrangement.

The potential window, ΔE, between the Zn potentials however, appears to be ~ 20 mV greater in this arrangement most likely due to the increased distance between the ORR electrode and the Zn anode and possibly the presence of the OER electrode in between. This is evidenced by values in Table S5 which indicate a greater zinc oxidation overpotential in this configuration, while zinc reduction overpotentials remained the same. The polarisation, ΔE, between ORR and OER at 20 mA cm^−2^ was steady at approx. 700 mV for the first 30 cycles, and the cell exhibited an energy efficiency of around 61%. Subsequently, the ΔE between ORR and OER increased by 20 mV every 10 cycles giving energy efficiencies of 58–60% between cycle 31 and 75. The increase in polarisation observed was mainly due to the increase of ORR overpotential as the OER electrode potential was stable between 0.556 and 0.565 V *vs.* Hg/HgO throughout the duration of the test. The ORR potential eventually reached − 0.663 V *vs.* Hg/HgO by cycle 90 whereby the test was discontinued.

Commercial ORR and OER electrodes were assessed under the same conditions as well (Fig. S8). The QSI-Nano commercial ORR electrode performance degraded rapidly in compressed air reaching exceedingly negative potentials after the first cycle. Additionally, although the mixed metal oxide OER electrode from Magneto displayed more consistent performance over this period of time, the OER overpotential was much greater than the Ni-Fe-Co(OH)_2_ on SS mesh electrode.

Previous studies (Table S7) on tri-electrode secondary Zn–air batteries report similar energy efficiencies between 50 and 60% at 20 mA cm^−2^. Dai et al.^19^ demonstrated a rechargeable Zn–air battery with a voltage polarisation of 0.70 V at 20 mA cm^−2^. Their battery utilised 6 M KOH + 0.2 M zincate at 20 to 50 mA cm^−2^ for ≥ 200 h in total with cycle periods of 4–20 h, reporting an energy efficiency of 65% at 20 mA cm^−2^. In this work, we used an electrolyte of lower molarity, 4 M NaOH + 0.3 M ZnO and obtained a slightly lower voltage polarisation performance of 0.69 V at 20 mA cm^−2^ and an initial energy efficiency of 61% which is promising in comparison to previous studies.

**Figure 9:**
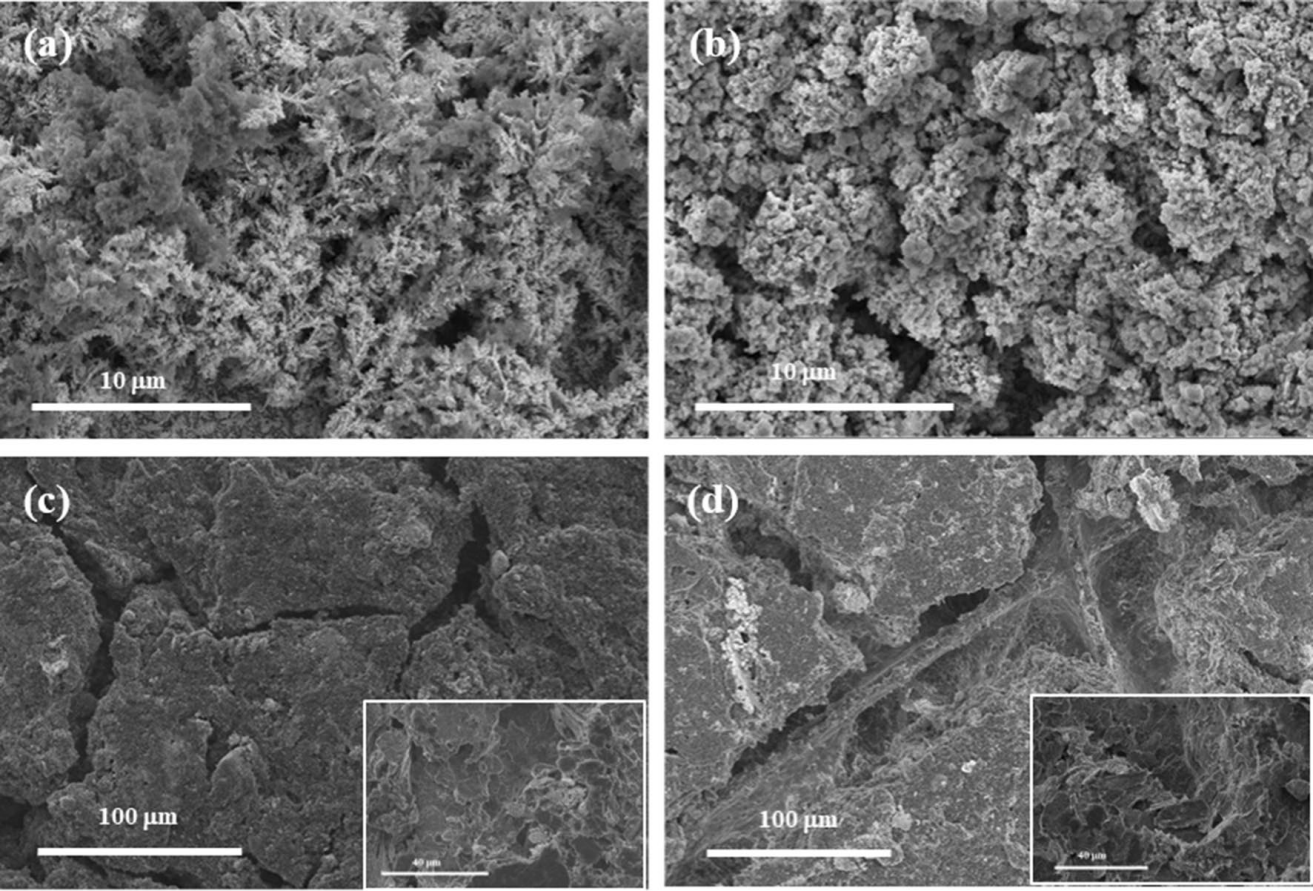
SEM images of Ni-Fe-Co(OH)_2_ coated SS mesh electrode (OER) (**a**) before cycling and (**b**) after cycling. MnO_x_ on carbon paper electrode (ORR) (**c**) before cycling and (**d**) after cycling; images of the back of the ORR electrode are displayed in the insets.

The MnO_x_ on C paper and Ni-Fe-Co(OH)_2_ on SS mesh electrodes were examined with SEM before and after cycling to see if there were any morphological differences in the catalyst layers. SEM images of the OER electrode before and after cycling in Fig. 9a,b respectively show no observable loss of catalyst layer. Contrary to this, there are noticeable changes in the MnO_x_ ORR catalyst layer on C paper after cycling. In Fig. 9d, the fibres of the carbon paper substrate are visible after cycling when compared to the SEM micrograph of the ORR electrode before cycling in Fig. 9c, which indicates that the catalyst layer had degraded during the long-term cycling test. The SEM images of the back of the ORR electrode (insets in Fig. 9c,d) which were facing the O_2_ supply did not display any apparent differences before and after cycling. It is therefore likely that one of the reasons for the increase in ORR overpotential after 75 cycles was due to the loss of catalyst layer from the carbon paper electrode over time as well as a gradual loss of PTFE and therefore hydrophobicity, resulting in flooding of the electrode which reduced the number of active sites for ORR. There is also a possibility that dissolution of MnO_x_ occurred^58^, however this was not monitored here.

Overall, the Ni-Fe-Co(OH)_2_ on SS mesh OER electrode demonstrated excellent stability over the whole testing period, whilst the MnO_x_ on C paper ORR electrode demonstrated adequate stability over 70 cycles in compressed air due to the composition and structure of the catalyst layer which helped to delay the onset of flooding.

## Conclusion

The selection of a suitable ORR catalyst was undertaken by studying the performance of in-house synthesised single and mixed metal oxide catalysts, carbon materials XC-72R, graphene and N-doped graphene and benchmark catalyst 5 wt% Pt/C. Our findings revealed that the catalysts can be grouped based on their *E*_*1/2*_ potentials: (I) metal oxides, (II) carbon materials and (III) precious metals. Further analysis of the data using the K-L equation gave an indication of the catalysts preference for the two or four electron ORR pathway. From the K-L plots, the slope values for MnO_x_ and MnCo_2_O_4_ were close to that of Pt/C signifying that the 4e^−^ pathway is dominant at these catalysts. The remaining catalysts ranked according to their absolute slope values were NiCo_2_O_4_ > N-doped graphene > MnO_2_ > XC-72R > Co_3_O_4_ ≈ graphene. Improvements in performance by the addition of Vulcan XC-72R was dependent on initial ORR activity. The GDE of MnO_x_ combined with XC-72R demonstrated highest ORR activity, with good stability over the range of current densities, indicating its suitability for further optimisation.

The optimised MnO_x_ GDE was tested in a secondary tri-electrode Zn–air cycling set-up and it was found that the MnO_x_ on C paper ORR electrode is sensitive to changes in temperature and current density whilst the Ni-Fe-Co(OH)_2_ on SS mesh OER electrode is sensitive to changes in electrolyte molarity. The cell was cycled at 20 mA cm^−2^ for a period of 50 h in compressed air with the ORR and OER electrodes displaying good performance and reasonable energy efficiencies between 58 and 61% for 40 h. The loss in energy efficiency from cycle 75 onwards was a result of the degradation in ORR electrode performance, observed from SEM images to be linked to the loss of catalyst layer and hydrophobicity over time, leading to premature flooding of gas diffusion pathways. Further improvements on the construction of the ORR catalyst layer is recommended to prolong stability.

## Supplementary Information

Supplementary Information.
