# Supplementary material for: Selection of oxygen reduction catalysts for secondary tri-electrode zinc–air batteries
Source: Sci Rep. 2022 Apr 23;12:6696. doi: 10.1038/s41598-022-10671-5 (PMC9035146; doi:10.1038/s41598-022-10671-5)
Supplement: Supplementary file 1 — Supplementary Information. [file 41598_2022_10671_MOESM1_ESM.docx]

**Supplementary Information**

**Selection of oxygen reduction catalysts for secondary tri-electrode zinc-air batteries**

Adeline Loh^1^, David P. Trudgeon^1^, Xiaohong Li^1*^, Mao-Cheng Liu^2^, Ling-Bin Kong^2^, Frank C. Walsh^3^

^1^ Renewable Energy Group, College of Engineering, Mathematics and Physical Sciences, University of Exeter, Penryn Campus, Cornwall TR10 9FE, UK

^2^ State Key Laboratory of Advanced Processing and Recycling of Non-Ferrous Metals, School of Materials Science and Engineering, Lanzhou University of Technology, Lanzhou 730050, China

^3^ Electrochemical Engineering Laboratory, Energy Technology Group, Department of Mechanical Engineering, University of Southampton, SO17 1BJ, UK.

* Corresponding author. Email: [X.Li@exeter.ac.uk](mailto:X.Li@exeter.ac.uk) Tel: 0044 (0)1326 255769

**Table S1** Advantages and disadvantages of secondary oxygen electrode designs.

|  | **Advantages** | **Disadvantages** |
| --- | --- | --- |
| Single-layer bifunctional catalyst | - Comparatively simple assembly - Cost-effective | - Bifunctional catalyst must be stable in full potential range - Ancillary additives (e.g. ionomers, carbon powders) may not be stable at oxidative potentials - Carbonate precipitation will affect the bifunctional catalyst layer |
| Dual or multi-layer catalyst | - Catalysts can be optimised for separate reactions - Suitable porosity can be achieved for ORR - Electrolytic contact between the two layers may be reduced during cycling | - Complex assembly - Potential mass transfer losses - Delamination or separation of layers by the pressure of the oxygen bubbles during evolution - Carbonate precipitation will affect both ORR and OER layers |
| Tri-electrode configuration | - Flexible design - Catalysts and electrodes can be optimised for separate reactions - Catalysts and ancillary additives need not be stable in full potential range | - Complex assembly which may add weight/ volume |

**Table S2** Corresponding performance data from cycle 10 of graphs in **Fig. 7a**

| *j*/ mA cm^-2^ | Charge / V | | | Discharge / V | | | Δ E / mV | | Energy efficiency / % |
| --- | --- | --- | --- | --- | --- | --- | --- | --- | --- |
|  | E_cell_ | E_we_ *vs* Hg/HgO | E_ce_ *vs* Hg/HgO | E_cell_ | E_we_ *vs* Hg/HgO | E_ce_ *vs* Hg/HgO | ORR and OER | Zn |  |
| 10 | -1.903 | 0.532 | -1.371 | 1.281 | -0.070 | -1.351 | 606 | 20 | 67 |
| 20 | -1.926 | 0.551 | -1.375 | 1.238 | -0.108 | -1.346 | 659 | 29 | 64 |
| 50 | -1.956 | 0.571 | -1.385 | 1.140 | -0.204 | -1.344 | 775 | 41 | 58 |
| 100 | -2.011 | 0.596 | -1.415 | 0.978 | -0.346 | -1.324 | 942 | 91 | 49 |

**Table S3** Corresponding performance data from cycle 10 of graphs in **Fig. 7b**

| Electrolyte molarity / M | Charge / V | | | Discharge / V | | | Δ E / mV | | Energy efficiency / % |
| --- | --- | --- | --- | --- | --- | --- | --- | --- | --- |
|  | E_cell_ | E_we_ *vs* Hg/HgO | E_ce_ *vs* Hg/HgO | E_cell_ | E_we_ *vs* Hg/HgO | E_ce_ *vs* Hg/HgO | ORR and OER | Zn |  |
| 2 | -1.944 | 0.590 | -1.354 | 1.195 | -0.126 | -1.321 | 716 | 33 | 62 |
| 4 | -1.926 | 0.551 | -1.375 | 1.238 | -0.108 | -1.346 | 659 | 29 | 64 |
| 6 | -1.927 | 0.535 | -1.392 | 1.247 | -0.117 | -1.364 | 652 | 28 | 65 |
| 8 | -1.939 | 0.525 | -1.414 | 1.241 | -0.140 | -1.381 | 665 | 33 | 64 |

**Table S4** Corresponding performance data from cycle 10 of graphs in **Fig.7c**

| Temperature / ºC | Charge / V | | | Discharge / V | | | Δ E / mV | | Energy efficiency / % |
| --- | --- | --- | --- | --- | --- | --- | --- | --- | --- |
|  | E_cell_ | E_we_ *vs* Hg/HgO | E_ce_ *vs* Hg/HgO | E_cell_ | E_we_ *vs* Hg/HgO | E_ce_ *vs* Hg/HgO | ORR and OER | Zn |  |
| 25 | -1.993 | 0.604 | -1.389 | 1.159 | -0.189 | -1.348 | 793 | 42 | 58 |
| 40 | -1.955 | 0.575 | -1.380 | 1.200 | -0.147 | -1.347 | 722 | 33 | 61 |
| 60 | -1.926 | 0.551 | -1.375 | 1.238 | -0.108 | -1.346 | 659 | 29 | 64 |
| 80 | -1.904 | 0.526 | -1.378 | 1.260 | -0.094 | -1.354 | 620 | 24 | 66 |

**Table S5** Corresponding performance data from cycle 10 of graphs in **Fig. 7d**

| O_2_ purity | Charge / V | | | Discharge / V | | | Δ E / mV | | Energy efficiency / % |
| --- | --- | --- | --- | --- | --- | --- | --- | --- | --- |
|  | E_cell_ | E_we_ *vs* Hg/HgO | E_ce_ *vs* Hg/HgO | E_cell_ | E_we_ *vs* Hg/HgO | E_ce_ *vs* Hg/HgO | ORR and OER | Zn |  |
| Bottled O_2_ | -1.926 | 0.551 | -1.375 | 1.238 | -0.108 | -1.346 | 659 | 29 | 64 |
| Air | -1.925 | 0.553 | -1.372 | 1.180 | -0.164 | -1.344 | 718 | 28 | 61 |
| Air (alt. arrangement) | -1.934 | 0.560 | -1.374 | 1.192 | -0.135 | -1.327 | 695 | 47 | 62 |

**Table S6** Conductivities of selected NaOH electrolyte concentrations

| **Electrolyte** | **Conductivity / S cm^-1^** |
| --- | --- |
| 2 M NaOH + 0.3 M ZnO | 0.266 |
| 4 M NaOH + 0.3 M ZnO | 0.353 |
| 6 M NaOH + 0.3 M ZnO | 0.362 |
| 8 M NaOH + 0.3 M ZnO | 0.369 |

**Table S7** Performance of tri-electrode secondary Zn-air systems in literature.

| **Cathode** | **Anode** | **Electrolyte** | **Cycling conditions and stability** | **Voltage polarisation at 20 mA cm^-2^ / V** | **Ref.** |
| --- | --- | --- | --- | --- | --- |
| ORR: MnO_2_ on C paper  OER: NiFeCo hydroxide coated SS mesh | Zn foil | 4 M NaOH + 0.3 M ZnO, *static* | - 10-50 mA cm^-2^, 30 min cycle periods for 50 h - 58-61 % energy efficiency at 20 mA cm^-2^ | 0.69 at 20 mA cm^-2^ | This work |
| ORR: CoO or MnO_2_  OER: 316 L SS mesh | 3D metal foam current collector filled with Zn, TiN and polymer binder | Commercial electrolyte mainly consisting of 7 M KOH, *static* | - 5-15 mA cm^-2^, 24-30 h cycle periods for ~120 h (4 cycles) - 50 % energy efficiency | > 0.90 at 5-15 mA cm^-2^ | [4] |
| ORR: CoO/ N-CNT on carbon fibre paper  OER: NiFe LDH on Ni foam | Zn foil | 6 M KOH + 0.2 M zincate, *static* | - 20-50 mA cm^-2^, 4-20 h cycle periods for > 200 h (10 cycles) - 65 % energy efficiency at 20 mA cm^-2^ | 0.70 at 20 mA cm^-2^ | [5] |
| ORR: macro/ meso-NC-NH_3_  OER: Co_3_O_4_ on Ni foam | Zn foil | 6 M KOH + 0.2 M ZnCl_2_, *static* | - 10 mA cm^-2^, 4 h cycle periods for ~800 h (200 cycles) - 60-65 % voltage efficiency at 10 mA cm^-2^ | 0.75 at 10 mA cm^-2^ | [6] |
| ORR: Commercial  GDE  OER: RuO_2_ on Ti net | Zn coated C felt on porous Cu foam | 6 M KOH + 0.4 M ZnO, *static* | - 20 mA cm^-2^, ~2 h cycle periods for 1917 h (1000 cycles) - 40 % energy efficiency | 1.1 V to 1.4 V at 20 mA cm^-2^ | [7] |
| ORR: Ag-based catalyst  OER: Ni foam | Cu foam soldered on Zn (not in contact with electrolyte) | 30 wt% KOH + 2 wt% ZnO, 30ºC, *flowing* 0.4 L min^-1^ | - 50 mA cm^-2^, ~8 min cycle periods, (>600 cycles) with short circuits - ~58 % voltage efficiency at 20 mA cm^-2^ | 1.2 V at 50 mA cm^-2^ (0.75 V at 20 mA cm^-2^) | [8] |


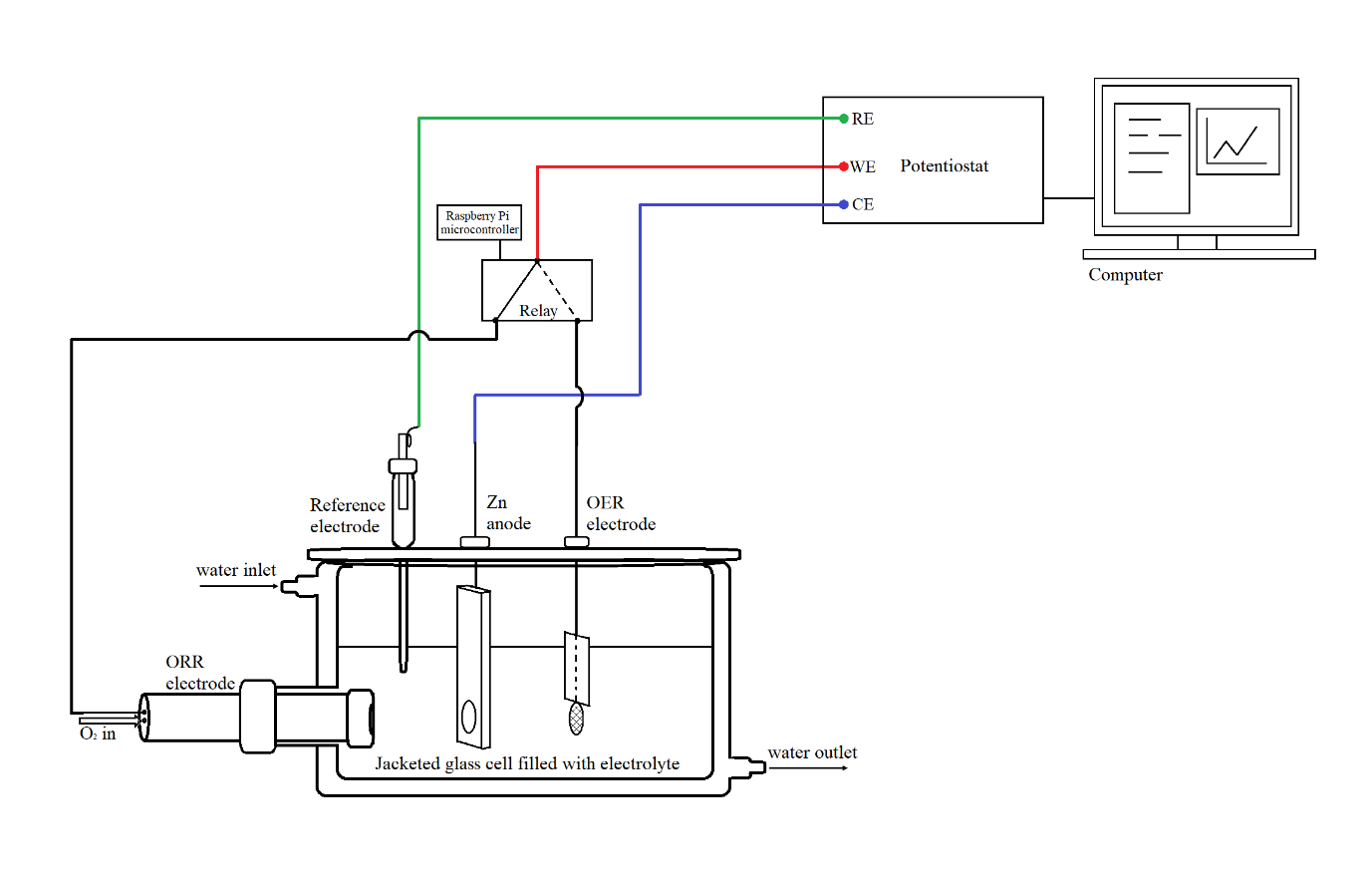


**Figure S1** Schematic illustration of electrodes in a jacketed glass cell, connected to Raspberry Pi-microcontroller and relay for galvanostatic cycling.

**(a)**

**(b)**

**Figure S2** Tafel slope plots for various ORR catalysts (a) without XC-72R and (b) with XC-72R, in O_2_ saturated 1 M NaOH at 298 K taken from LSVs measured at 400 rpm and scan rate of 5 mV s^-1^.

**(a)**

**(b)**

**Figure S3** Koutecky-Levich plots j^-1^ versus ω^-1/2^ for (a) catalysts and **(**b) catalysts with Vulcan XC-72R taken from the mass transfer controlled region of -0.4 V for rotation rates 100, 400, 900, 1600 and 2500 rpm.


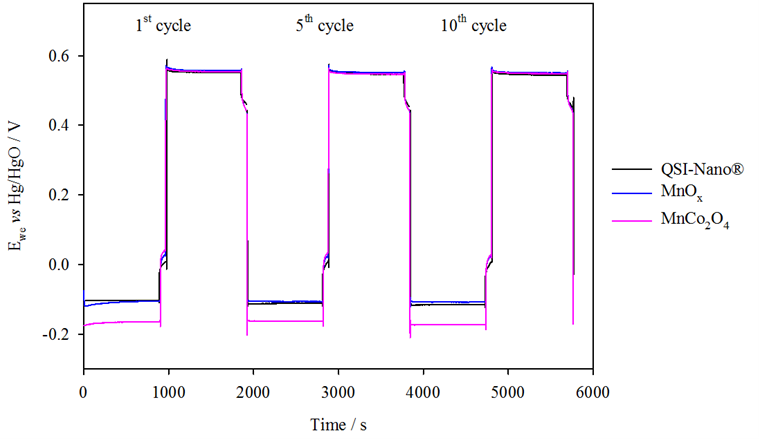


**Fig. S4** Comparison of ORR electrodes at 20 mA cm^-2^ in static electrolyte 4 M NaOH + 0.3 M ZnO at 333 K and O_2_ flow rate 200 cm^3^ min^-1^.


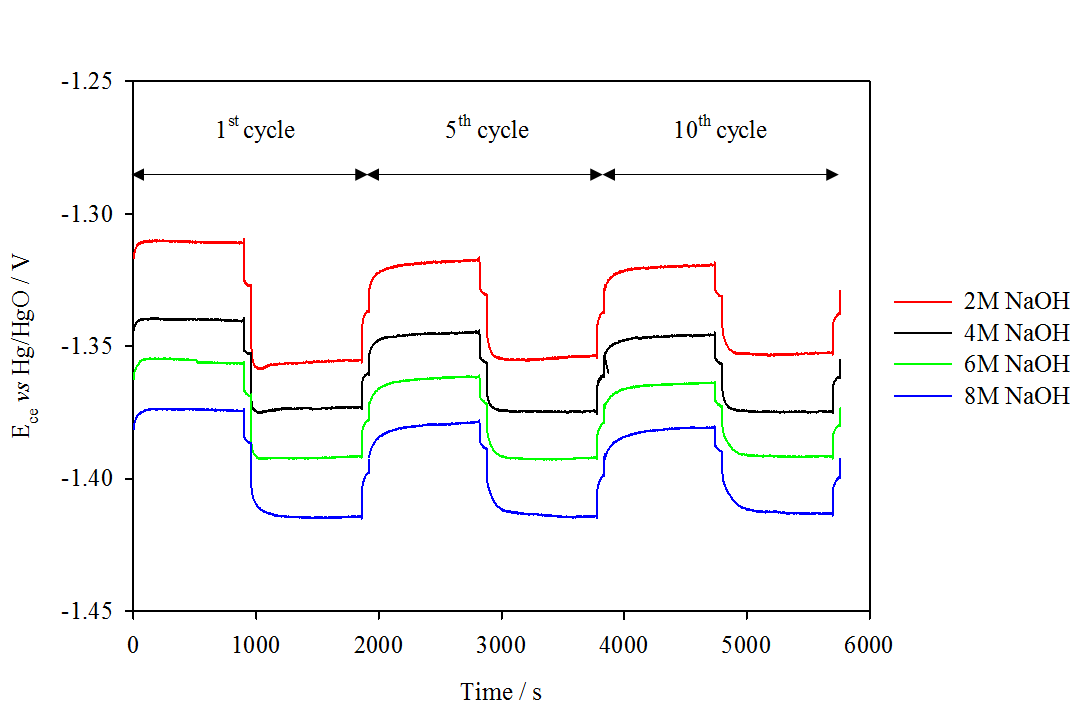


**Fig. S5** Comparison of Zn electrode performance at 20 mA cm^-2^ in static electrolyte of varying NaOH concentration. A constant concentration of 0.3 M ZnO was added to the electrolyte and experiments were run at 333 K.

**
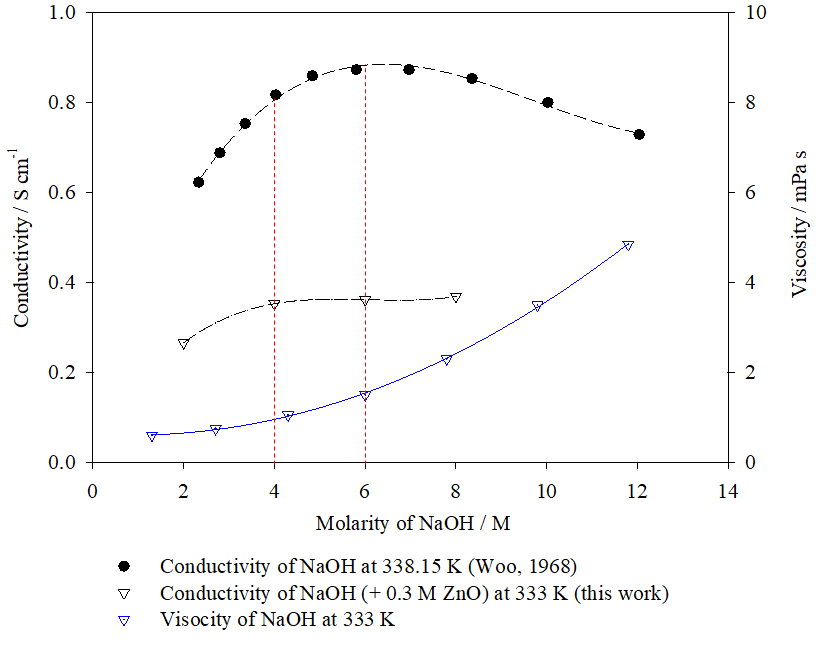
Fig. S6** Comparison of the conductivity [1] and viscosity [2] of NaOH over a range of concentrations at 333 K.

The reported conductivities of different NaOH molarities at 338.15 K are much greater than that of the NaOH + 0.3 M ZnO electrolytes measured in this work which indicates that the addition of ZnO reduces the conductivity [3]. In **Fig. S6**, conductivities of 4 M NaOH + 0.3 M ZnO and 6 M NaOH + 0.3 M ZnO electrolytes at 333 K are observed to be similar at around 0.36 S cm^-1^, however the viscosity of 6 M NaOH is much greater than 4 M NaOH.

**Fig. S7** Changes in overpotential at the ORR and OER electrode versus (a) current density, (b) electrolyte molarity and (c) temperature.

**(a) (b)**

**(c)**

**Fig. S8** Galvanostatic cycling performance of MnO_x_ + Ni-Fe-Co(OH)_2_ electrodes (―) and commercial QSI-Nano® ORR and Magneto OER electrodes (**―**) in a tri-electrode Zn-air set-up over 50 h at 20 mA cm^-2^ with 30 min cycle periods, in static electrolyte of 4 M NaOH + 0.3 M ZnO at 333 K. Compressed air was supplied at 200 cm^3^ min^-1^.

**References**

1. Woo, A.L.W. *Conductance studies of concentrated solutions of sodium hydroxide and potassium hydroxide electrolytes*, in *Chemistry*. South Dakota State University: South Dakota, USA (1968).

2. O'Brien, T.F., Bommaraju, T.V. & Hine, F. Handbook of Chlor-Alkali Technology. Boston, MA (2005).

3. Liu, M.B. *et al.* Conductivity of KOH electrolyte supersaturated with zincate. *J. Electrochem. Soc.* **128**, 2049-52. <https://doi.org/10.1149/1.2127187> (1981).

4. Toussaint, G. *et al.* Development of a rechargeable zinc-air battery. *ECS Trans.* **28**, 25-34. <https://doi.org/10.1149/MA2010-01/12/757> (2010).

5. Li, Y. *et al.* Advanced zinc-air batteries based on high-performance hybrid electrocatalysts. *Nat. Commun.* **4,** 1805. <https://doi.org/10.1038/ncomms2812> (2013).

6. Li, L. *et al.* Hierarchical pore-in-pore and wire-in-wire catalysts for rechargeable Zn– and Li–air batteries with ultra-long cycle life and high cell efficiency. *Energy Environ. Sci.* **8**, 3274-82. <https://doi.org/10.1039/C5EE02616D> (2015).

7. Hong, W., Li, H. & Wang, B. A horizontal three-electrode structure for zinc-air batteries with long-term cycle life and high performance. *Int. J. Electrochem. Sci.* **11**, 3843-51. <https://doi.org/10.20964/110384> (2016).

8. Bockelmann, M., Kunz, U. & Turek, T. Electrically rechargeable zinc-oxygen flow battery with high power density. *Electrochem. Commun.* **69**, 24-7. <https://doi.org/10.1016/j.elecom.2016.05.013> (2016).
